# Biomechanical analysis of fixation methods in acetabular fractures: a systematic review of test setups

**DOI:** 10.1007/s00068-022-01936-9

**Published:** 2022-03-19

**Authors:** Nico Hinz, Julius Dehoust, Matthias Münch, Klaus Seide, Tobias Barth, Arndt-Peter Schulz, Karl-Heinz Frosch, Maximilian J. Hartel

**Affiliations:** 1 Department of Trauma Surgery, Orthopedic and Sports Traumatology, BG Hospital Hamburg, Bergedorfer Strasse 10, 21033 Hamburg, Germany; 2 Laboratory for Biomechanics, BG Hospital Hamburg, Bergedorfer Strasse 10, 21033 Hamburg, Germany; 3 Fraunhofer Research Institution for Individualized and Cell-Based Medical Engineering, Mönkhofer Weg 239 a, 23562 Lübeck, Germany; 4 Department of Trauma and Orthopedic Surgery, University Medical Center Hamburg-Eppendorf, Martinistrasse 52, 20246 Hamburg, Germany

**Keywords:** Acetabular fracture, Biomechanical analysis, Finite element analysis, Fracture fixation, Clinical implications, Inter-comparability

## Abstract

**Purpose:**

Optimal anatomical reduction and stable fixation of acetabular fractures are important in avoiding secondary dislocation and osteoarthritis. Biomechanical studies of treatment options of acetabular fractures aim to evaluate the biomechanical properties of different fixation methods. As the setup of the biomechanical test can influence the experimental results, this review aimed to analyze the characteristics, comparability and clinical implications of studies on biomechanical test setups and finite element analyses in the fixation of acetabular fractures.

**Methods:**

A systematic literature research was conducted according to the PRISMA guidelines, using the PubMed/MEDLINE and Web of Science databases. 44 studies conducting biomechanical analyses of fixation of acetabular fractures were identified, which met the predefined inclusion and exclusion criteria and which were published in English between 2000 and April 16, 2021. The studies were analyzed with respect to distinct parameters, including fracture type, material of pelvis model, investigated fixation construct, loading direction, loading protocol, maximum loading force, outcome parameter and measurement method.

**Results:**

In summary, there was no standardized test setup within the studies on fixation constructs for acetabular fractures. It is therefore difficult to compare the studies directly, as they employ a variety of different test parameters. Furthermore, the clinical implications of the biomechanical studies should be scrutinized, since several test parameters were not based on observations of the human physiology.

**Conclusion:**

The limited comparability and restricted clinical implications should be kept in mind when interpreting the results of biomechanical studies and when designing test setups to evaluate fixation methods for acetabular fractures.

**Supplementary Information:**

The online version contains supplementary material available at 10.1007/s00068-022-01936-9.

## Introduction

Acetabular fractures have an incidence of approximately 3 patients/100,000/year [1] and mostly occur in two distinct age groups: young patients develop acetabular fractures after high energy trauma, whereas elderly patients develop acetabular fractures after low energy trauma associated with osteoporotic bone status [2–5].

The standard procedure for displaced and unstable acetabular fractures consists of surgical anatomical reconstruction, followed by internal fixation or total hip arthroplasty (THA) [2, 3, 6–10]. An accurate anatomical reduction and biomechanically stable fixation remains a challenge in the management of acetabular fractures because of the complex geometry, the limited fracture exposure, and the need of a fixation of both columns in fracture types involving the anterior and posterior columns [6, 11, 12]. Although the rate of surgically treated acetabular fractures is steadily increasing, perfect anatomical fracture reduction is achieved in only 64% of cases [13]. Approximately 18–40% of patients who underwent surgical fixation of an acetabular fracture develop posttraumatic osteoarthritis and thus require secondary THA after a mean interval of 22–42 months between initial surgery and THA [6, 9, 10, 12, 14–16]. The main predictors for posttraumatic osteoarthritis are not only non-accurate anatomical reduction during surgery but also loss of anatomical reduction during the postoperative period as a consequence of a biomechanically unstable fixation of the fracture [6, 12, 16–20].

Thus, biomechanical studies of different fixation methods are important to determine optimal fixation techniques and test novel implants in a preclinical setting. Furthermore, biomechanical and pre-clinical analysis of surgical implants is required according to the ISO 14602 standard to take all variables, which can influence the intended performance of the new implant, under consideration [21]. However, several setup variables should be considered when performing a biomechanical comparison of osteosynthesis techniques. In this context, the optimal test setup should incorporate parameters (e.g. loading type, outcome variables), which essentially map physiological properties in vivo and can thus generate clinically relevant and realistic data [22]. A standardized test protocol could improve the comparability of biomechanical studies and ensure that the test setup mimics the processes in vivo.

Therefore, this review aimed to summarize the available studies reporting a biomechanical assessment of different fixation methods for acetabular fractures. The issues in this systematic literature review can be summarized in the following questions: What kind of test setup parameters were primarily used? Was there a standardized biomechanical test protocol? Did the test protocols mimic the physiological properties in humans and thus provided clinically relevant and realistic data?

## Materials and methods

This review was focused on biomechanical studies evaluating plate, screw, THA and/or other internal fixation methods in different acetabular fractures using cadaveric pelves, synthetic pelves, or finite element analysis (FEA). Therefore, a systematic literature review was conducted according to the Preferred Reporting Items for Systematic Review and Meta-analysis (PRISMA) guidelines as specified in the PRISMA 2020 statements and checklist (Supplementary Table 3) [23, 24]. The complete review protocol is presented below.

The PubMed/MEDLINE database and the Web of Science Core Collections database were used on April 16, 2021 to identify appropriate publications. The basic search terms were *biomechanical testing*, *biomechanical comparison*, *biomechanical analysis*, *biomechanical investigation*, *biomechanical evaluation*, and *biomechanical study* linked by a Boolean *OR*. The basic search terms were combined with *acetabulum fracture OR acetabular fracture* using a Boolean *AND* to limit the research to the specific anatomical region. A total of 484 studies were identified during the literature search of the two databases. The publications were further screened for eligibility by one reviewer (NH), who applied the following inclusion and exclusion criteria within the title and abstract:

Inclusion criteria:

1. Biomechanical studies investigating plate, screw, THA and/or other internal fixation methods for human acetabular fractures
2. Studies using human cadaveric pelves, synthetic pelves or FEA
3. Studies published between 2000 and April 16, 2021

Exclusion criteria.

1. Biomechanical studies on external fixation methods
2. Biomechanical studies on primary implantation of total hip endoprosthesis without an acetabular fracture or revision surgery of acetabular fractures e.g. acetabular discontinuity with revision THA
3. Biomechanical studies on methods of fixation in other anatomical regions than those specified above, for example fractures of the pelvis ring, and femoral head or neck
4. Studies on postoperative treatment analysis, clinical investigations, clinical case reports or animal experiments
5. Studies without a biomechanical test setup, finite element analysis or investigation of a fixation method
6. Studies which were not available in English
7. Repeated items found during the search process

**Fig. 1:**
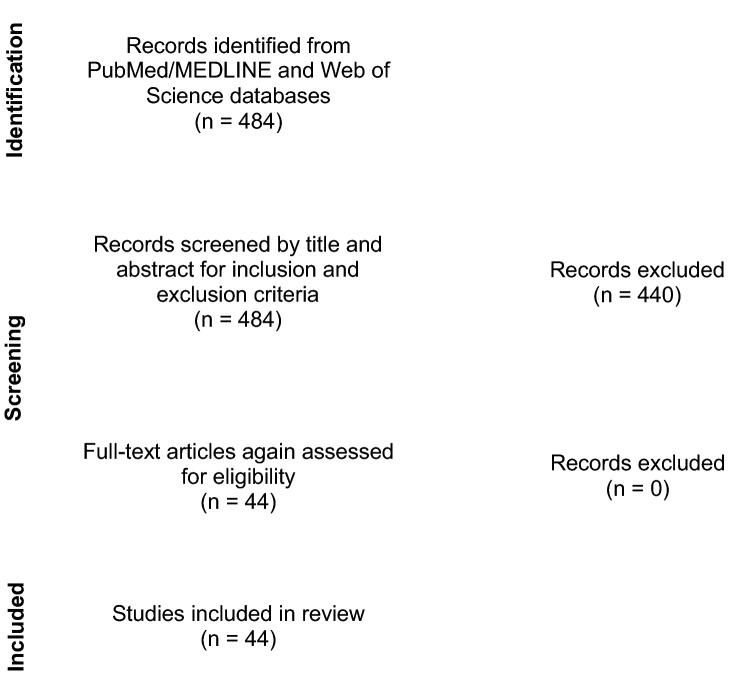
Schematic illustration of the article selection process (modified from PRISMA 2020 flow diagram)

Finally, 44 studies were identified which fulfilled the inclusion criteria. No additional studies were excluded after the full texts were analyzed with respect to the inclusion and exclusion criteria. Figure 1 schematically portrays the article selection process.

The subsequent data extraction was primarily performed by one reviewer (NH) in consultation with the whole team of authors to check the plausibility of the decisions. All articles were analyzed for the investigated fracture type and were assigned to the following groups according to the Judet and Letournel classification: posterior column/wall fracture, anterior column fracture, transverse fracture, T-shaped fracture, anterior column posterior hemitransverse fracture (ACPHT), or associated both column fracture [25, 26]. If one study investigated more than one fracture type, the biomechanical analysis was assigned to all investigated fracture types and the test setup was examined separately for each fracture type. In a second step, the biomechanical analyses were assigned to two categories: biomechanical test setups using physical tests or FEA using simulations with the finite element method. All reported biomechanical test setups were further examined using the following categories and secondary characteristics:

- Material type of the used pelvis model: synthetic pelvis; cadaveric pelvis.
- Fixation method that was biomechanically tested: plate osteosynthesis (non-locking or locking); screw osteosynthesis (not primarily used within plate osteosynthesis); THA (cemented or uncemented); others (e.g. cable fixation or other internal fixators).
- Direction of the loading force applied during biomechanical testing: single-leg stance; single-leg stance with mobile pelvis; double-limb stance; mediosuperior direction (45° mediosuperiorly and 15°–25° posteriorly); direction perpendicular to acetabulum; sitting; sit-to-stand; others/not specified.
- Loading protocol according to the classification of Schorler et al. [27] and the maximum loading force: quasi-static loading; cyclic loading; load to failure.
- Method of measuring the outcome variables: optical measurement system; ultrasound-based system; mechanical (digital) distance indicator; integrated displacement sensor; strain gauge; beam sensor; pressure-sensitive film.
- Measurement parameter used for the biomechanical comparison of different methods of fixation: displacement [mm; µm; °]; stiffness [N/mm]; force/cycles at construct failure [N; n]; failure energy [N*cycles; J]; stress distribution [MPa]; contact area, load and stress distribution within acetabulum [cm^2^; N; MPa]; yield and maximum strength [MPa]; others (elastic energy [kJ]; yield force [N]; plastic energy [kJ]; maximum force [N]; deformation [με]).

All publications using an FEA were further analyzed by the following categories and secondary characteristics:

- Investigated fixation method: plate osteosynthesis (non-locking or locking); screw osteosynthesis (not primarily used within plate osteosynthesis); THA (cemented or uncemented); others (e.g. cable fixation).
- Direction of the loading force applied during simulated biomechanical analysis: double-limb stance; single-leg stance; sitting; sit-to-stand; climbing stairs; mediosuperior direction (45° mediosuperiorly and 20° posteriorly); others.
- Loading protocol of simulated loading and the maximum loading force: static loading; cyclic loading.
- Calculated measurement parameter used for the biomechanical comparison of different methods of fixation: displacement [mm]; stiffness [N/mm]; von Mises stress distribution [MPa]; others.

The secondary characteristic are defined in detail in the results section, since some inconsistencies require a more detailed description. If a test setup was not described clearly enough in the original publication or did not fit perfectly into one of the defined groups, it was assigned to the best fitting group—as described in detail in the results section. If one characteristic of a test setup was not specified at all, it was not assigned to any group. The cases of unclearly defined or not defined test setup characteristic were discussed by the whole reviewer team until consensus was obtained. As some of the publications used more than one test method and thus investigated more than one fracture type, measurement method or outcome variable, the total number of detected secondary characteristics differ from the total number of articles found.

**Fig. 2:**
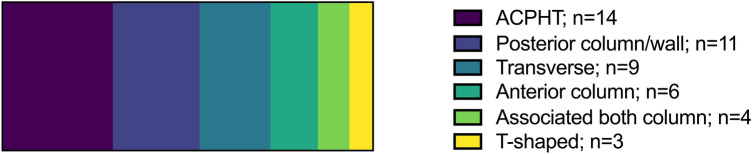
Numbers of different types of acetabular fractures reported in the studies analyzed (biomechanical test setups and FEA are summarized)

**Fig. 3:**
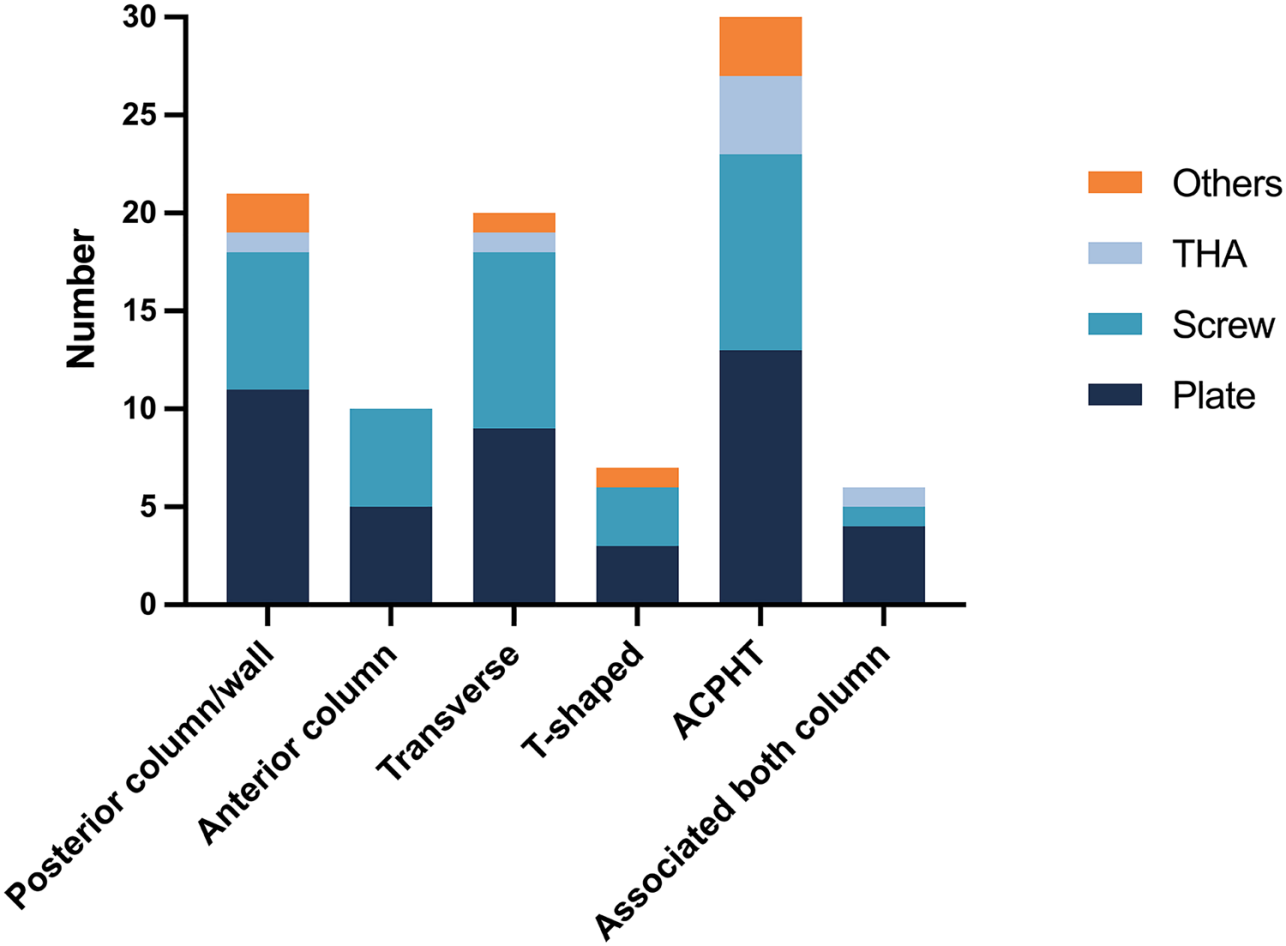
Frequency distribution of investigated fixation methods, summarized for biomechanical test setups and FEA, and grouped by fracture types

**Fig. 4:**
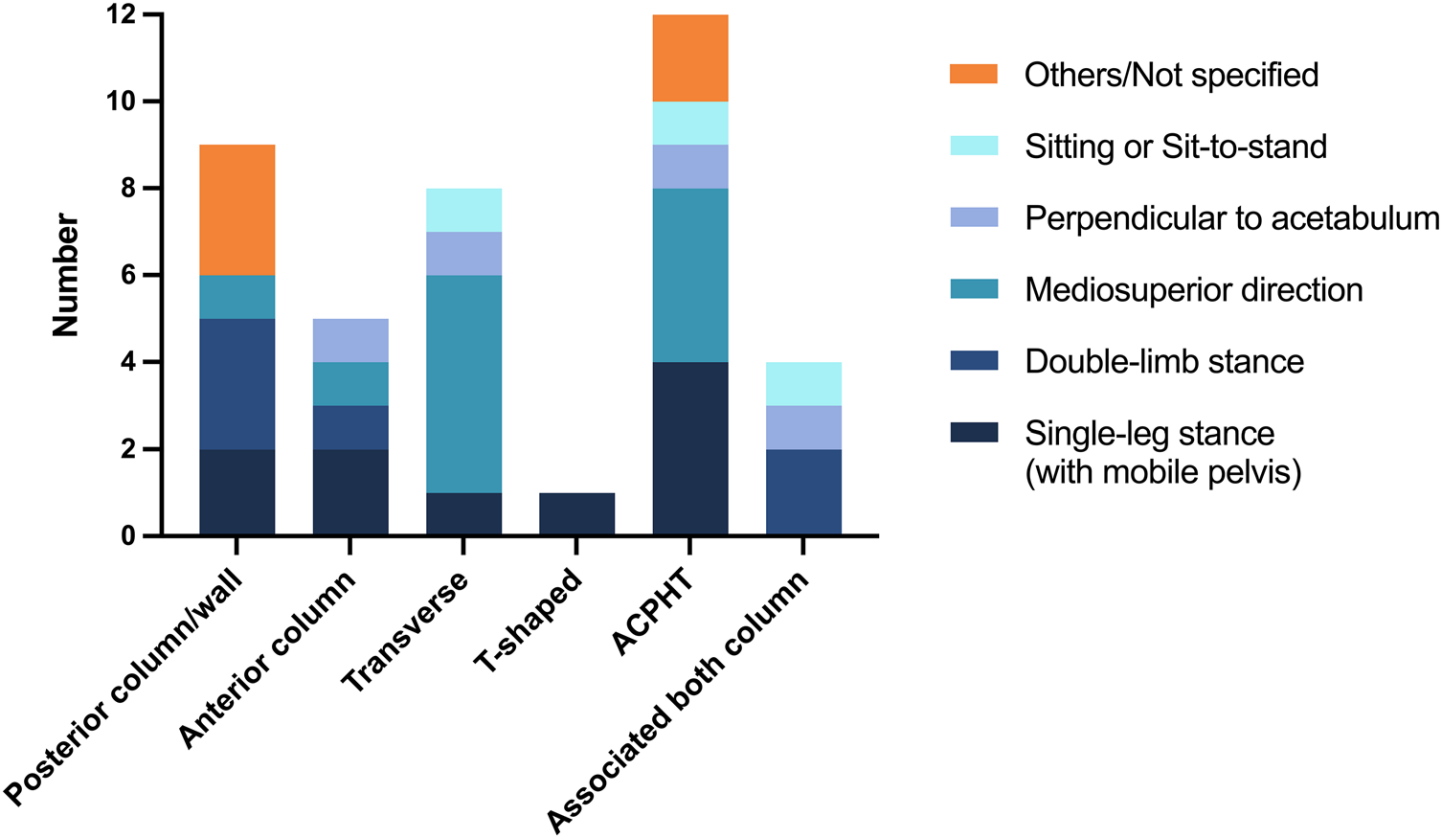
Frequency distribution of biomechanical test setups using different loading directions, grouped by fracture types

**Fig. 5:**
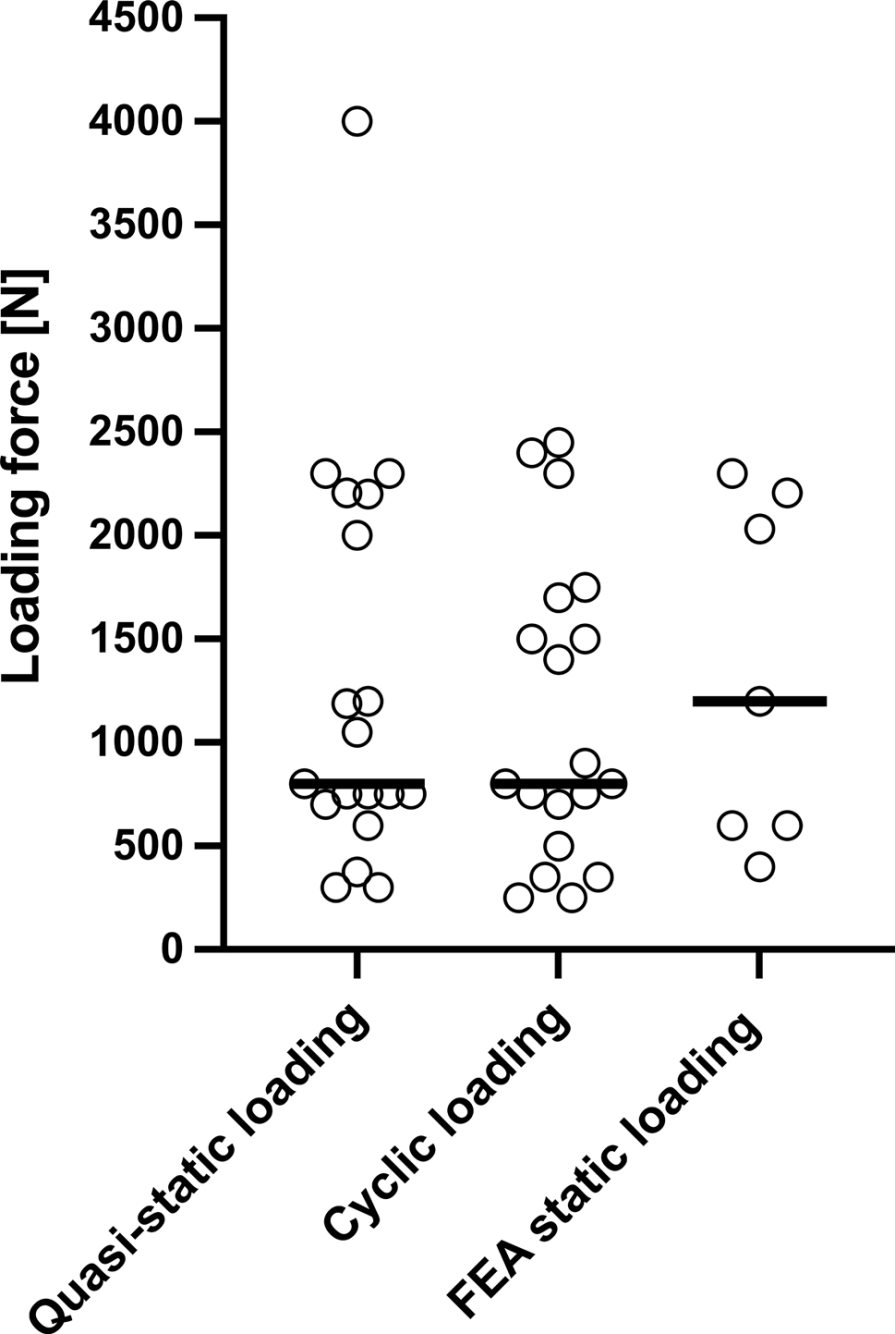
Scatter plot of maximum loading forces in *N* applied during quasi-static loading (*n* = 19), cyclic loading (*n* = 19), and FEA static loading (*n* = 7). Single dots represent values of individual studies. Thick lines represent medians

The numbers of biomechanical test setups and finite element analyses using the distinct characteristics were summarized in tables grouped by the corresponding fracture types. GraphPad Prism 9 software was used to create Figs. 2, 3, 4 and 5 and to determine the medians of all maximum loading forces reported within the publications using biomechanical test setups as well as FEA. Studies, which did not specify the applied loading force or only provided split joint forces within a coordinate system, were not included in Fig. 5 and in the determination of medians. If a study applied the same loading force in the analysis of more than one fracture type, this loading force was included only once in the determination of medians. Medians of maximum loading forces were not compared statistically and are intended for illustrative purposes only.

## Results

Of the initial 484 studies found during the systematic literature research, 44 studies met the inclusion and exclusion criteria and were further analyzed according to the above-mentioned categories and secondary characteristics (Fig. 1). The frequency distributions of the secondary characteristics for biomechanical test setups are displayed in Table 1 (for more detail see Supplementary Table 1) and for FEA in Table 2 (for more detail see Supplementary Table 2). The frequency distributions in Table 1 and 2 are grouped by the type of acetabular fracture investigated.

**Table 1:** Analysis of studies using a biomechanical test setup for evaluation of fixation constructs in acetabular fractures

	Posterior column/wall fracture	Anterior column fracture	Transverse fracture	T-shaped fracture	Anterior column posterior hemitransverse fracture	Associated both column fracture	Total
Total number	9	5	7	1	11	3	36
*Material type of pelvis model*							
Synthetic pelves	4	4	5	1	11	–	25
Cadaveric pelves	5	1	2	–	1	3	12
*Fixation methods investigated*							
Plate	9	4	7	1	10	3	34
Screw	5	4	7	1	8	1	26
THA	1	–	1	–	3	–	5
Others	2	–	1	1	2	–	6
*Loading direction*							
Single-leg stance	2	–	1	1	3	–	7
Single leg stance with mobile pelvis	–	2	–	–	1	–	3
Double-limb stance	3	1	–	–	–	2	6
Mediosuperior direction or comparable directions	1	1	5	–	4	–	11
Perpendicular to acetabulum	–	1	1	–	1	1	4
Sitting	–	–	–	–	–	1	1
Sit-to-stand	–	–	1	–	1	–	2
Others/not specified	3	–	–	–	2	–	5
*Loading protocol [max. loading force]*							
Quasi-static loading	3 [2200 N–4000 N]	2 [300 N–2300 N]	3 [750 N–2000 N]	1 [600 N]	7 [300 N–2207 N]	2 [700 N–800 N]	18
Cyclic loading	6 [350 N–2300 N]	3 [750 N–800 N]	3 [250 N–1750 N]	1 [250 N]	5 [350 N–2450 N]	1 [700 N]	19
Load to failure	1	3	5	–	4	1	14
Measurement method							
Optical measurement system	5	2	6	–	8	1	22
Ultrasound-based system	1	2	–	1	1	–	5
Mechanical (digital) distance indicator	1	–	–	–	1	2	4
Integrated displacement sensor	2	1	1	–	1	–	5
Strain gauge	1	–	1	–	–	–	2
Beam sensor	–	–	–	–	–	2	2
Pressure-sensitive film	2	–	–	–	–	–	2
*Outcome parameter*							
Fracture displacement and femoral head displacement	8	5	6	1	11	3	34
Stiffness	5	2	6	1	5	3	22
Force/cycles at construct failure	1	3	4	–	4	1	13
Failure energy	–	1	–	–	2	–	3
Stress distribution	1	–	1	–	–	–	2
Yield and maximum strength	–	–	1	–	–	–	1
Contact area, load and pressure distribution within acetabulum	2	–	–	–	–	–	2
Others	1	2	–	–	–	–	3

The frequency distributions are grouped the fracture types. The numbers of biomechanical test setups with the corresponding items are displayed in the table cells

**Table 2:** Analysis of studies using a FEA for evaluation of fixation constructs in acetabular fractures. The frequency distributions are grouped by the fracture types. The numbers of FEA with the corresponding items are displayed in the table cells

	Posterior column/wall fracture	Anterior column fracture	Transverse fracture	T-shaped fracture	Anterior column posterior hemitransverse fracture	Associated both column fracture	Total
Total number	2	1	2	2	3	1	11
*Fixation method investigated*							
Plate	2	1	2	2	3	1	11
Screw	2	1	2	2	2	–	9
THA	–	–	–	–	1	1	2
Others	–	–	–	–	1	–	1
*Loading direction*							
Double-limb stance	1	1	1	1	1	1	6
Single-leg stance	1	–	–	–	–	–	1
Sitting	–	–	1	–	–	–	1
Sit-to-stand	1	–	–	–	–	1	2
Climbing stairs	–	–	–	–	–	1	1
Mediosuperior direction	–	–	–	–	1	–	1
Others	–	–	1	1	1	–	3
*Loading protocol [max. loading force]*							
Static loading	1 [1200 N]	1 [2300 N]	2 [400 N–2032 N]	2 [600 N–2032 N]	3 [600 N–2207 N/complex]	1 [n/a]	10
Cyclic loading	1 [900 N]	–	–	–	–	–	1
*Outcome parameter*							
Fracture displacement	2	1	2	2	2	1	10
Stiffness	–	–	–	1	1	–	2
von Mises Stress distribution	1	1	2	2	2	1	9
Others	–	–	1	1	–	–	2

In summary, 36 biomechanical test setups were identified that consisted of a synthetic or cadaveric pelvis model [28–63]. In these pelvis models, distinct acetabular fractures were created and then fixed with different methods of osteosynthesis and/or THA. Subsequently the prepared pelves were mechanically loaded with a wide range of loading protocols and directions. Additionally, 11 FEA were identified, in which pelvis models with distinct acetabular fractures were virtually generated [62–71]. Following the simulation of osteosynthetic fixation and/or THA, a virtual load was applied using different loading protocols. Two studies performed a biomechanical test as well as an FEA [62, 63].

### Fracture classification

Acetabular fractures were osteotomized in the physical pelves or simulated in the FEA by taking into consideration the typical fracture classification of Judet and Letournel [25]. The most frequently investigated fracture types in biomechanical tests as well as in FEA were an ACPHT fracture (biomechanical tests: 11; FEA: 3) [36–44, 60, 63, 66, 71], a posterior column/wall fracture (biomechanical tests: 9; FEA: 2) [52–59, 62, 67], and a transverse acetabular fracture (biomechanical tests: 7; FEA: 2) [28–34, 70]. Other fracture types (associated both column fracture, T-shape fracture and isolated anterior column fracture) were less frequently studied in biomechanical analyses [35, 45–51, 61, 64, 65, 68–70]. The numbers of investigated fracture types within the reported studies are demonstrated in Fig. 2—summarized for biomechanical test setups and FEA.

### Material types of pelvis model

For biomechanical tests, research groups could choose between human cadaveric pelves prepared in various ways, or synthetic pelvis models, which consisted of bone substitutes intended to reflect the biomechanics of physiological bone. Most research groups investigating fixation methods for acetabular fractures used synthetic pelves models (25 test setups) [28–32, 35–44, 49–51, 54–56, 60–63]. These models consisted of a whole pelvis or a hemipelvis model fixed in different ways to enable particular loading directions. Only 32% (12 test setups) of the pelvis models were human cadaveric pelves [33, 34, 44–48, 52, 53, 57–59]. One study even used a synthetic as well as a cadaveric pelvis model [44]. Cadaveric pelves and synthetic pelves were used about equally often for testing posterior wall fractures (synthetic models: 4; cadaveric models: 5), but in other fracture types synthetic pelvis models were used more frequently. Abnormalities of the cadaveric pelvis were ruled out by radiography, DXA and/or CT scan. The human pelves were prepared in different ways, either by removing the femur and loading with an artificial femoral head or by maintaining both proximal femora and embedding them for double-limb stance loading. Furthermore, soft tissues, such as ligaments of the SI-joint or hip joint capsule, were removed to a variable extent in the different publications.

### Fixation methods investigated

Figure 3 portrays the frequency distribution of the different fixation methods, summarized for biomechanical test setups and FEA, and grouped by fracture types.

Since osteosynthesis of acetabular fractures with plates and screws is the standard surgical treatment, the most frequently investigated fixation methods were plate and screw osteosynthesis constructs in biomechanical test setups (Plate: 34; Screw: 26) [28–59, 61–63] as well as in FEA (Plate: 11; Screw: 9) [63–71]. Anterior column plates, posterior columnplates, anterior column screws and posterior column screws were common osteosynthesis constructs, which were biomechanically studied for transverse acetabular fractures. Most studies on the fixation of ACPHT fractures used anterior column plates (suprapectineal plate, infrapectineal plate, pelvic brim plate), quadrilateral buttress plates, posterior column plates, posterior column screws and anterior column screws. Reconstruction plates and lag screws were mainly compared for posterior wall fractures, whereas different anterior column plates and screws were compared for fixation of isolated anterior column fractures. Most of the investigated plate systems had non-locking plate-screw connections, and only a minority of the studies evaluated a locking plate system. Furthermore, seven biomechanical analyses assessed the biomechanical properties of a THA either alone or in combination with other fixation methods [29, 42, 53, 60, 63, 68]. THAs were mostly analyzed in studies dealing with posterior wall fractures, transverse fractures and ACPHT fractures. A few studies investigated other fixation methods: cable/wire fixation [34, 63], a subcutaneous internal anterior fixation [35], an acetabular fracture reduction internal fixator [43], an acetabular tridimensional memory alloy-fixation system [58] or calcium phosphate cement [59].

### Loading directions

The directions of the loading force in the biomechanical analyses reviewed were assigned to seven main groups: single-leg stance, single-leg stance with a mobile pelvis, double-limb stance, mediosuperior direction, direction perpendicular to the acetabulum, sitting position and sit-to-stand loading. Figure 4 portrays the distribution of the number of biomechanical test setups using different loading directions, grouped by fracture types.

A simple single-leg stance loading direction was used in 7 of 36 biomechanical test setups and consisted of an anatomical femoral hip prosthesis, which was mounted with the shaft and moveably connected with the pelvis model in a single-leg stance position, usually with 15° anteversion [28, 35, 36, 39, 42, 55, 62]. The loading force was applied via defined parts (e.g. SI-joint or sacrum) of the (hemi-)pelvis model. Culemann et al. established a modified single-leg stance model with a mobile pelvis, which was fixed on the head of a hip prosthesis with cord units simulating the pull forces of the hip abductor muscles. The axial compressive load was applied through the first sacral vertebra via a ball joint. This allows the pelvis to move in all three planes [44]. Comparable models of a modified single-leg stance protocol were applied in a total of four biomechanical tests [44, 50, 51].

A double-limb stance protocol was mainly applied with a cadaveric pelvis model and in studies evaluating posterior wall fractures or associated both column fractures (6 biomechanical test setups) [45, 48, 52, 57, 58, 61]. In a double-limb stance model, both proximal femoral shafts of the human cadaveric pelves or synthetic pelves were rigidly fixed and the loading force was applied through the fourth lumbar vertebrae with the pelvis in a specific neutral position [45, 48, 52, 57, 58, 61].

In one commonly used loading protocol, the loading force was applied with an artificial femoral head (without a simulated CCD angle of an anatomical femoral hip prosthesis) in a mediosuperior direction of approximately 45° mediosuperiorly and 15° to 25° posteriorly (12 biomechanical test setups) [29–32, 34, 37, 38, 41, 49, 53, 63]. Such a loading direction was frequently used for the evaluation of transverse fractures and ACPHT fractures. The model of a mediosuperior loading direction was based on observations of Bergman et al., who evaluated hip joint loading during daily activities by implanting telemetering total hip prosthesis in patients undergoing THA. They observed that the main loading force on the acetabulum during the stance runs along the axis of the femoral neck with a mediosuperior angle of 45° [72–74]. Mehin et al. applied the loading force with an artificial femoral head perpendicularly to the acetabulum, in order to provoke movements at the fracture sites [33]. This loading perpendicular to the acetabulum was found in four biomechanical test setups [33, 43, 46, 47].

A few research groups extended their experiments with sitting or sit-to-stand loading. To test the pelvis model under sitting conditions, Wu et al. mounted a human cadaveric pelvis in a sitting position as described by Gao et al. and axially loaded it through the fourth lumbar vertebrae [48, 75]. In addition, two biomechanical tests investigated fixation methods transverse and ACPHT fractures using a sit-to-stand loading protocol [28, 36]. For this purpose, the pelves were mounted, for example, with 28° posterior pelvic tilt and were axially loaded from the SI-joint with a hip flexion of 95° with a femoral hip prosthesis.

A few research groups developed test setups which could not be assigned to one of the groups and were therefore assigned to the category “others”. For instance, Becker et al. further improved the model of a single-leg stance with mobile pelvis as described by Culemann et al. by additionally using an adjustable wire with a load cell to tilt the pelvis model into a specific position, and thus achieved loading angles comparable to those in the in vivo observations of Bergmann et al. [60, 76]. Pease et al. mounted a synthetic pelvis so that the anterior superior iliac spine and symphysis pubis were vertical, and the joint surface of the symphysis was vertical at 90° to this plane. Additionally, flexion of the pelvis in the hip joint was simulated with the pelvis loaded axially by an artificial femoral head [54]. Olson et al. used a single-leg stance protocol, in which a linear actuator applied the loading force via a simulated abductor mechanism to a cadaveric pelvis rigidly mounted at the sacrum and moveably connected to the femur [59]. Some other studies did not exactly specify their test setups within their manuscript [40, 56].

Studies reporting a FEA of fixation methods for acetabular fractures mainly used a loading protocol with a double-limb stance (6 FEA) [64–69]. Some of the studies also evaluated additional loading forms, such as a loading in a sitting position [64], sit-to-stand loading with different angles of hip flexion [67, 68], or loading that simulated stair climbing [68]. Aziz et al. combined a biomechanical testing approach with a subsequent FEA. Consequently, they used a protocol in the FEA similar to their biomechanical test protocol with the aforementioned loading direction of 45° mediosuperiorly and 20° posteriorly [63]. In their study on fixation methods for transverse and T-shaped fractures, Terzini et al. specified that the resultant force of their FEA was orientated according to the reaction forces observed by Bergman et al. in vivo [70]. Liu et al. used different loading directions in a multi-part model, which were based on the resultant forces during different gait phases, as observed by Bergman et al. in vivo. The direction of loading varied between 22° hip flexion and 14° hip extension [71].

### Loading protocols and loading forces

Three different loading protocols were distinguished in the biomechanical test setups: quasi-static loading, cyclic loading and loading to failure. The loading protocols were categorized and defined according to the considerations and definitions of Schorler et al. [27]. A quasi-static loading was defined as a non-repeated, continuously increasing application of a loading force up to a predefined maximal force. In contrast, a cyclic loading protocol was defined as a repeated application of a predefined loading force—at least with more than one loading cycle with a sinusoidal load pattern. The loading force could be either increased or consistent during the cyclic application. 18 of 36 biomechanical tests performed a quasi-static loading protocol, with a maximum force of 300 N–4000 N (median: 800 N) [28, 29, 32, 35, 36, 39, 41–47, 55, 58, 59, 61, 63]. In comparison, a cyclic loading protocol was reported in 19 of 36 biomechanical test setups with a maximum force of 250 N–2450 N (median: 800 N) [30, 31, 33, 35, 37–40, 48–57, 60, 62]. The number of cycles applied during cyclic loading varied greatly between the studies. The spectrum ranged from 5 cycles up to 42,000 cycles. Some of the research groups maintained the same loading force during the cyclic loading whereas other studies applied more complex protocols, in which increasing forces simulated different phases of the postoperative period. In studies evaluating posterior column/wall fractures or anterior column fractures, a cyclic loading protocol was more prevalent, whereas a quasi-static loading protocol was more frequently used in the analysis of ACPHT fractures.

In 14 of 36 biomechanical tests a loading to failure protocol was performed [29–31, 33, 34, 38, 40, 43, 46, 47, 49, 51, 56, 63], which means that the pelves were continuously loaded with an increasing force or with cyclic loading until a predefined failure criterion was reached, such as material fracture, dislocation of the femoral head or displacement of the fracture sites beyond specified values.

Among the studies using FEA, 10 of 11 FEA applied a static loading protocol with forces between 400 and 2300 N (median: 1200 N) [63–71]. One study using FEA applied a multi-part static loading protocol with different loading forces simulating resultant forces of different gait phases [71]. One FEA simulated a cyclic loading protocol with a force up to 900 N. The number of cycles was not specified in this manuscript [62].

To obtain an overview of the commonly used loading forces in the single loading protocols, the maximum loading forces applied during quasi-static loading, cyclic loading and in FEA static loading were identified and medians were determined. The maximum loading forces in *N* applied during quasi-static loading, cyclic loading and in FEA static loading are presented in Fig. 5, as reported in all analyzed studies.

### Outcome parameters and measurement methods

Displacement was the main outcome parameter, which was measured during 34 of 36 biomechanical tests [28–33, 35–57, 59–63]. Many types of displacement were evaluated during biomechanical testing, and were mostly reported in mm or °. The parameter gap motion was defined as the absolute enlargement of the fracture gap, whereas gap sliding was specified as the shift of the fragments against each other along the fracture line. In contrast, the parameter interfragmentary motion was defined as a change in the distance between different fracture fragments and/or to the intact pelvis. Displacement parameters used less frequently included the longitudinal motion of the pelvis or vector dislocation. Interfragmentary rotation was defined as the rotational component between the fracture fragments in °. Beside these parameters, some studies investigated a displacement or rather subluxation of the femoral head transmitting the load.

The displacement parameters were measured by various methods. An optical measurement system was most frequently selected to determine displacement and was applied in 22 of 36 biomechanical test setups [28–33, 36–41, 46, 49, 53–56, 60–63]. This method used passive markers, which were attached to the pelves, osteosynthesis constructs and/or the load transmitting component of the testing machine. Both position and relative movements of the markers were usually determined by high precision cameras. Other methods that were used less frequently for the evaluation of displacement, were an ultrasound-based system in five biomechanical test setups (sensor pairs consisting of an ultrasonic microphone and an ultrasonic reader) [35, 44, 50, 51, 57], mechanical (digital) distance sensors in four biomechanical test setups (mechanical indicator measuring changes in distance using analogue or digital displays) [42, 45, 48, 59] and a beam sensor in two biomechanical test setups (sensor pair determining the relative distance via a photoelectric effect) [45, 48]. A few experiments measured the subluxation of the load transmitting femoral head with integrated displacement sensors of the biomechanical testing machine (5 of 34 biomechanical test setups) [34, 43, 47, 52, 53]. Most studies that determined the fracture displacement also calculated the construct stiffness, defined as the force per unit length in N/mm, by means of a linear regression (22 of 36 biomechanical tests) [29–35, 37, 39, 41, 43, 45–49, 52, 54–57, 63].

To compare the load to failure of different fixation methods, the loading force and/or the number of cycles at the point of construct failure were recorded during 13 of 36 biomechanical tests [29–31, 33, 38, 40, 43, 46, 47, 49, 51, 56, 63]. The studies specified mechanical failure criteria, such as a sudden loss of force resistance or a significant change in the stress–strain curve, and/or clinical failure criteria, such as fracture displacement of more than 2–3 mm, femoral head displacement of more than 5–10 mm, (sub-) luxation of the femoral head or (out-)breakage of the osteosynthesis construct. In three biomechanical tests, the failure energy was additionally calculated, which was defined as the total area under the force/displacement curve in N*cycles or in J [38, 49, 63].

A few studies evaluated additionally outcome variables. For instance, Chang et al. and Su et al. investigated the stress distribution [MPa]/deformation [με] of the pelves/osteosynthesis constructs under loading by using strain gauges [34, 52]. Chang et al. further calculated the yield and maximum strength of the fixation constructs [34]. Xin-Wei et al. and Olson et al. determined the contact area [cm^2^] as well as load [N] and pressure [MPa] distribution within the acetabulum by positioning a pressure sensitive film between the femoral head and the acetabulum of human cadaveric pelves [58, 59]. Only a few studies evaluated elastic energy [kJ], yield force [N], plastic energy [kJ] maximum force [N] and mode of failure during load to failure tests [47, 49].

There was less variety in the outcome variables determined in FEA studies. 10 of 11 FEA calculated the displacement (along the fracture line or as interfragmentary motion in mm) [62, 64–71] and 9 of 11 FEA calculated the von Mises Stress distribution [MPa] of the pelves/osteosynthesis constructs under virtual loading [63–70]. In two FEA, the construct stiffness was calculated from the force/displacement curve [65, 66], and in two FEA the axial and shear strain were calculated from the displacement values [70].

## Discussion

The objective of this review was to analyze biomechanical test setups and FEA used to evaluate different fixation methods for acetabular fractures. During the systematic literature review the following questions were addressed: was there a standardized or at least commonly used test setup? Did the biomechanical experiments mimic the physiological properties and were they therefore able to generate clinically relevant and realistic results?

### Considerations on fracture classification and fixation methods

Simple posterior column/wall fractures, complex anterior column fractures with a posterior hemitransverse component and associated both column fractures are among the most common types of acetabular fractures [5, 10, 77, 78]. It can be assumed, that the high incidence of these fractures has enhanced their priority within the research community, resulting in more biomechanical studies. Consistently, the most frequently investigated fracture types in the biomechanical studies reviewed were an ACPHT fracture and a posterior column/wall fracture. Another reason why ACPHT fractures and other fractures involving both columns, e.g. transverse fractures, were frequently investigated in biomechanical studies may be that the complex anatomy of these fractures results in a challenge for adequate reduction and fixation. An alternative to the commonly used double plate fixation of both columns is to combine a plate osteosynthesis of one column with a screw osteosynthesis of the opposite column [13, 79–81]. This raised the question whether plate osteosynthesis combined with lag screw fixation is biomechanically equivalent to double plate osteosynthesis of both columns, and subsequently, which kind of combined plate/screw fixation is biomechanically superior. Consequently, these questions were frequently addressed by the biomechanical studies reviewed.

In surgical treatment of displaced and unstable acetabular fractures, plate and screw osteosyntheses remain the gold standard [2, 5, 82–84]. Additionally, various acetabular fracture types can be treated with distinct osteosynthesis constructs and combinations through different surgical approaches, which are steadily being improved [85]. Thus, the high prevalence of screw and plate osteosynthesis in biomechanical studies demonstrates the clinical relevance of these treatment options and the necessity of biomechanical characterization of the steadily growing range of options.

Another clinically relevant question relates to the option of a THA to treat dislocated acetabular fractures. Especially in elderly patients with preexisting degenerative arthritis, the lacking possibility of a restricted weight-bearing, high grade of acetabulum impaction/comminution, or accompanying fracture of the femoral head or neck, primary THA is recommendable and yields good clinical outcomes in comparison to osteosynthetic fixation [15, 84, 86]. Because of its clinical relevance, THA was the third most abundant fixation method analyzed in the biomechanical studies.

A few biomechanical studies investigated other rare or new fixation methods, such as cable fixation, INFIX (subcutaneous internal anterior fixation) system, AFRIF (acetabular fracture reduction internal fixator), ATMF (acetabular tridimensional memory alloy-fixation) system, or calcium phosphate cement. Such innovative methods are usually examined in preclinical biomechanical studies before their feasibility and clinical outcome in humans is evaluated.

### Considerations on material types of pelvis model and FEA

Most biomechanical studies analyzed during this review used synthetic bone substitutes. Only a few research groups employed human cadaveric pelvis models. The key arguments in favor of using a synthetic pelvis model for biomechanical studies are stated in the following: firstly, synthetic pelves assure standardized biomechanical conditions and geometry. This reduces confounding variables due to individual bone quality and structure, as well as anatomical characteristics of cadaveric bone, and thereby increases comparability and reproducibility [22, 29–32, 35, 38, 43, 44, 46, 49–51, 54–56, 58, 62, 63, 87]. Secondly, experiments with fresh frozen human pelves are more prone to error. For example, a higher non-cooling time was shown to lead to lower screw pullout strength [87, 88]. Thirdly, synthetic pelves are more easily available and are cheaper than cadaveric pelves [22, 29, 50, 56].

In contrast, research groups using cadaveric pelves argue as follows: firstly, cadaveric pelves represent the structural, morphological, and mechanical properties of bone in vivo more nearly than do synthetic pelves. In particular, the osteoporotic bone quality of cadaveric pelves can reproduce the osteoporotic bone of elderly patients, who frequently suffer from acetabular fractures [22, 33, 37, 39, 40, 42, 47]. Although bone substitutes mimicking the osteoporotic bone qualities are already available [22, 87], they were rarely used in the studies analyzed. Secondly, the influence of soft tissues, such as ligaments, muscles and the labrum, was to some extent included in the assessment [28, 37, 39, 40, 49, 56, 62, 63]. The periarticular soft tissues, the ligamentous capsule the cartilage and the labrum were shown to contribute significantly to the physiological kinematics and biomechanics of the hip joint [89–92]. However, a large proportion of the soft tissues, especially the muscles, was also removed during the preparation of cadaveric pelves. For this reason, Fensky et al. developed a table construction, which enables biomechanical testing of undissected cadaveric pelves by fixing and axially loading complete human cadavers [93].

Several studies targeting the mechanical validation of synthetic bone composite models (e.g. femur, tibia and humerus) revealed that synthetic bone substitutes exhibit a comparable biomechanical behavior of the bone itself when compared to human cadaveric bones [94–96]. Furthermore, in their standard F1839-08, the American Society for Testing and Materials recommends the use of rigid polyurethane foam blocks or sheets for mechanical testing of orthopedic devices [97]. Nevertheless, Girardi et al. identified significant differences in the strain magnitude and orientation between human cadaveric pelves and fourth generation composite pelves [98]. In addition, in load to failure tests of locking nail fixation Ziran et al. found that there were differences in the failure modes between cadaveric and synthetic proximal femora [99]. Consequently, the type of the pelvis model should be considered when interpreting the data of biomechanical studies. Moreover, the use of different pelves models and materials impairs the comparability between the studies.

The use of a FEA in studies is a well-established method. For instance, Anderson et al. found that cortical strain values in human cadaveric pelves and FEA, based on the computed tomography image data of the human pelvis, were highly correlated [100]. Nevertheless, it should be kept in mind that this method also incorporates assumptions and simplifications of the physiological biomechanical properties in humans.

### Considerations on loading directions

With their studies in 1993 and 2001, Bergmann et al. shaped the research on biomechanics of the hip joint. They examined the loading parameters and contact forces within the hip joint during walking, running and other daily activities, by using instrumented total hip implants with telemetric data transmission in patients. In this way, they showed that the main loading direction during the stance phase of gait is directed mediosuperiorly with about 45° and with a posterior angle of about 15° [72–74]. Therefore, several biomechanical research groups loaded the acetabulum with a femoral head in a 45° superomedial and 15° posterior direction. A distinction must be made between mediosuperior loading protocol and a single-leg stance loading protocol, which consists of an anatomical femoral hip prosthesis axially loaded to the acetabulum with about 15° anteversion. As stated above, the soft tissues (namely labrum, cartilage, ligaments and muscles) have a measurable influence on the biomechanics of the hip joint [89–92, 101]. As a consequence, if the acetabulum is loaded with an anatomical femoral hip prosthesis to simulate the proximal femur in a physiological stance position, this may lead to altered loading patterns of the acetabulum compared to the identified loading direction in the intact human pelvis with soft tissues as in Bergmann et al. [72, 73].

Culemann et al. refined the single-leg stance model by moveably fixing the pelvis on the head of a hip prosthesis with cord units, thus simulating the pull of the hip abductor muscles [44]. Becker et al. further improved this model by using an adjustable wire to change the tilt of the hemipelvis [60]. By doing so, they generated loading angles comparable to the resultant forces observed in vivo by Bergman et al. [72, 73]. Olson et al. also modified the single-leg stance model by indirectly loading the acetabulum of a moveably fixed cadaveric pelvis via a simulated abductor mechanism with a linear actuator [59]. These models addressed the importance of the abductor muscles for stabilization of the pelvis in the mediolateral plane during walking [101–103]. Although the modifications of Culemann et al. and Olson et al. tried to overcome the biomechanical problems of missing soft tissues, the exact resulting loading direction in these models remain unclear and can still differ from the physiological loading direction.

Other research groups used loading directions which are not based on the above-mentioned biomechanical characteristics of the hip joint. For instance, Mehin et al. observed no fracture motion in a transverse fracture model after loading the acetabulum in a direction of 45° superomedially and 15° posteriorly [33]. Consequently, they and other research groups applied a loading direction perpendicular to the acetabulum, in order to provoke motion at the fracture site. However, such a loading protocol may not represent physiological loading conditions, and thus the clinical implications of these studies should be treated with caution.

Loading protocols simulating sitting or the transition from a sitting to a standing position were underrepresented in the biomechanical studies analyzed. Nevertheless, sitting is a main activity of daily life and therefore sitting and sit-to-stand loading protocols should be implemented more often in biomechanical analyses [104–106]. During a sit-to-stand movement, the hip joint is exposed to an even higher peak pressure than in the stance position [72]. This can lead to overestimation of construct stability in studies which only perform stance loading protocols with a force representing body weight.

Studies using the FEA employed a wider range and complexity of loading directions than studies using a biomechanical test setup. Liu et al. simulated various loading directions based on the loading directions observed by Bergman et al. during different gait phases in vivo [71, 76]. In an FEA, Kocsis et al. applied a double-limb stance loading as well as a sit-to-stand and a climbing stairs loading in a FEA [68]. Furthermore, the hip joint forces differ during the various phases of the sit-to-stand movement, depending on the flexion angle of the hip [107, 108]. Thus, Lei et al. performed a sit-to-stand loading with various angles of hip flexion in their FEA [67]. Multiple loading forces depending on the different phases of the gait circle or of the sit-to-stance movement should be implemented into biomechanical test setups.

One has to keep in mind that the studies of Bergmann et al. identifying the loading direction of 45° superomedial and 15° posterior were based on the forces measured with the femoral component of a hip endoprosthesis, but not with the acetabular component [72, 73]. These studies of the proximal femur detected only minor changes in the loading direction during the gait cycle, even when the data were mathematically transformed for the resultant forces in the acetabulum [74]. However, using another mathematical model, Pedersen et al. observed a significant variation in the directions of acetabular contact forces during the gait cycle of about 12°–63° medially and 15°–50° posteriorly [109]. The FEA of Liu et al. considered these differing loading directions in their loading protocol [71]. Additionally, the contact areas of the acetabulum were found to depend on the loading force during walking. Light loading forces resulted in contact areas in the anterior and posterior acetabulum, whereas higher loads (about more than 50% body weight) led to loading of the acetabular dome [74, 110]. These variations in the force vector and contact area should be considered and implemented in biomechanical analyses of the acetabulum, since this can influence the investigated failure mode and the stiffness of fixation constructs in acetabular fractures.

As the biomechanics of the hip are complex and the experiments incorporate assumptions and simplifications, it is essential to be cautious when interpreting the data of the biomechanical studies and their clinical implications. Moreover, the loading directions differ widely between studies and are sometimes even not specified, so that it is difficult to compare the biomechanical studies and their results.

### Considerations on loading forces

A typical loading force used in a single leg stance or mediosuperior loading in the studies analyzed was 750 N and the median maximum forces used during quasi-static or cyclic loading were 800 N. In some studies, a loading force of 750 N was assumed to represent a full body weight of a 75 kg person in single leg stance position and accordingly a force of about 350 N was defined as partial weight bearing [29, 37, 40, 46, 49–51, 56, 60]. Based on these considerations, a maximum force of 600 N was frequently used as a loading force in FEA and defined as body weight [65–67]. Su et al. and Zhang et al. used a loading force of 1400 N (corresponding to about 2 times body weight) in their biomechanical double-limb stance protocols [52, 57].

It should be kept in mind that single leg stance is only one part of the gait circle. Several studies observed higher forces within the hip joint during the gait circle. Peak forces of about 238–324% of body weight at a walking speed of 3–4 km/h were measured in patients with telemetric hip endoprosthesis [72, 73, 111–113]. Mathematical calculations identified even larger average forces of 4–4.3 times the body weight during walking [112, 114, 115]. This is why, several of the studies reviewed applied higher loading forces of 1700 N to 2207 N (representing 2.5 to 3 times body weight) during single-leg stance or mediosuperior loading [30, 31, 54, 59, 63, 70]. Consistently, other studies specified 750 N as a loading force occurring during partial weight bearing with 50% of the body weight and 300 N as loading with partial weight bearing with 20 kg [28, 43, 47].

Some studies applied even higher loading forces (up to 2450 N) to simulate climbing stairs or stumbling, but without changing the loading direction [38]. In patients with telemetric hip endoprosthesis, loading forces up to 870% of body weight were measured during stumbling and mean peak forces of 11,000 N were calculated from these data [73, 113]. Studies investigating a sit-to-stand loading used a loading force up to 750 N. However, Bergmann et al. identified a peak force of 190% of body weight or rather an average peak force of 1500 N during standing up from a chair [72, 113]. Altun et al. and Kacira et al. referred to the ISO 7206-4 “Implants for surgery—Partial and total hip joint prostheses” when testing different plate osteosynthesis constructs with 2300 N [55, 61]. In contrast, several biomechanical studies used different loading forces (from 250 N up to 4000 N) without referring to any physiological conditions [32, 33, 35, 41, 44, 48, 58, 62, 64].

The broad spectrum of loading forces applied in the biomechanical test setups and the inconsistent specification of partial and full weight bearing forces again impairs the comparability between the studies. It should be noted that a biomechanical analysis with partial weight loading only mimics the early postoperative period. Furthermore, unexpected high loading forces due to stumbling can also occur in the postoperative period but were only included in a few studies. This may lead to overestimation of the biomechanical stability of the fixation constructs analyzed in several studies.

### Considerations on loading protocols

Quasi-static loading and cyclic loading protocols can have different clinical implications and, to our knowledge, a cyclic loading protocol can mimic the physiological loading more adequately than a quasi-static loading protocol [22, 116]. A few studies showed a higher strain rate and a greater biological effect during cyclic loading protocols than during static loading protocols [116–118]. Thus, a quasi-static loading may lead to overestimation of the stability of fixation constructs.

The cyclic loading protocols varied widely with respect to the number of cycles, ranging from 5 cycles up to 42,000 cycles. It should be taken into account that a classical fatigue cyclic loading protocol requires a high number of repetitions of about 100,000 or more [27]. The ASTM international defined a number of 1 million cycles as an appropriate cycle number for biomechanical testing of the endurance limit [87, 119]. No experiment analyzed in this review reached such a high cycle number. When calculating the cycles per year during walking in patients with a telemetric hip endoprosthesis, Bergmann et al. determined a median of 1,369,300 up to 2,553,400 cycles per year as a realistic load for normal and active patients, respectively. Furthermore, they calculated 41,400–140,200 cycles per year for climbing stairs and 20,100–64,600 cycles per year for sit-to-stand movements as realistic loading conditions for in vitro biomechanical testing of hip implants [113]. In contrast, Olson et al. concluded that 200,000 to 250,000 cycles are enough during a cyclic loading protocol to cover the time until a fracture is healed [87].

Some studies applied a constant loading force during the cyclic loading protocol [30, 33, 35, 39, 40, 48, 50–52, 55–57, 59, 62], whereas a few research groups applied more complex cyclic loading protocols with increasing loading forces, in order to simulate increasing loading forces during the postoperative period [31, 37, 38, 49, 53, 54, 60]. The latter protocols can reproduce the complexity of the postoperative period in humans more adequately, provided that appropriate loading forces were chosen. The high variation of the number of cycles and the application of simple or complex dynamic loading protocols limits the comparability between the studies.

Several studies performed a load to failure test. This type of biomechanical testing may not be clinically relevant, since unphysiological force values are reached during these tests, and thus clinically irrelevant modes of failure may occur. Consistently, Gardner et al. concluded in their review that supraphysiological loads from one direction during load to failure protocols are completely uncommon in the postoperative period [22]. Hence, load to failure tests are able to identify biomechanically superior fixation constructs on a theoretical level but their clinical implications should be interpreted with caution. Furthermore, the failure criteria were inconsistently defined among the studies. As a consequence, the force/number of cycles at failure are hard to compare between the studies.

### Considerations on outcome parameters

The main outcome parameters measured during the biomechanical studies were displacement [mm] and stiffness [N/mm]. These parameters are important and clinically relevant outcome variables in biomechanical studies, as displacement of the fracture sites due to a low construct stiffness can lead to osteoarthritis after acetabular fractures [9, 12, 16, 17, 20]. Nevertheless, the measurement of displacement and therefore the basis of the calculated stiffness was inconsistent and poorly defined in some studies. Most of the studies determined the gap motion or the interfragmentary motion between the fracture fragments or in relationship to the intact pelvis, whereas other studies evaluated interfragmentary rotation, interfragmentary sliding or subluxation of the femoral head. The inconsistency in definition of displacement variables can make it more difficult to compare the construct stiffness of the studies. Furthermore, the relationship between stiffness and fracture healing is an important issue in current research. A high stiffness of locked-plate osteosynthesis can impair fracture healing, because micromotion is necessary for the formation of callus [120]. Thus, it should be taken with caution to conclude that a fixation construct is biomechanically superior on the basis of high stiffness values.

Acetabular contact area and load and mean/peak pressure distribution within the acetabulum are other clinically relevant parameters in the studies reviewed, as they may represent cartilage stress. Alterations in cartilage stress can contribute to the development of posttraumatic osteoarthritis [103]. Many of the studies employ other biomechanical outcome parameters, e.g. stress distribution, mode of failure, force/cycles at construct failure, failure energy, elastic/plastic energy and yield/maximum force, which makes it more difficult to compare the study results. In contrast, nearly all studies using a FEA computationally determined the von Mises stress distribution, supporting the comparability of these studies.

### Considerations on measurement methods

In the biomechanical test setups, the great majority of measurement methods were an optical measurement system. These systems were proven to generate data up to the level of micrometers [121, 122]. Consequently, optical measurement systems offer a well-established and well-validated measurement system for biomechanical analyses of displacement, motions and for the calculation of construct stiffness.

Other measurement methods to evaluate displacement and stiffness are also well-established and validated within the biomechanical literature; this includes ultrasound-based systems, integrated displacement sensors of testing machines, strain gauges and beam sensors [123]. Pressure-sensitive films are commonly used to measure acetabular contact areas and pressure distribution within the acetabulum. These pressure transducers give a precise and dynamic output but their spatial resolution is low and the thickness of the film may influence hip biomechanics [103]. In sum, each measurement system possesses specific advantages and difficulties, which need to be born in mind when interpreting the study results.

### Comparison to other reviews

The absence of a standardized testing protocol among the biomechanical studies reviewed is in line with previous systematic reviews dealing with biomechanical tests of osteosynthesis constructs. In a large systematic review of 159 biomechanical studies on bone plate osteosynthesis in different anatomical locations, Schorler et al. concluded that no standardized test setup was used. They concluded that it is difficult to compare the different study results due to inconsistencies [27]. Moazen et al. systematically reviewed biomechanical studies of THA periprosthetic femur fracture fixation. They came to a similar conclusion that comparison between the studies was suboptimal [124].

### Limitations

Some limitations of this review must be reported. Firstly, screening of the publications for eligibility and data extraction was not performed independently in duplicate, due to reasons of time and personnel. However, the whole reviewer team checked the decisions for plausibility. Thus, it can be assumed that this limitation does not change the key statements and conclusions of this review.

Secondly, idealized groups for the categories fracture type, material type of pelvis models, investigated fixation method, loading direction, loading protocol, measurement method and measurement parameter were defined. Consequently, a few biomechanical testing protocols were categorized into the best fitting group, even though they contain additional information, for example on the structure of a gradually increasing loading force during the cyclic loading. However, biomechanically important aspects were not lost due to the categorization into idealized groups and the important facts were reported within the main text of the manuscript.

Thirdly, further details, such as the exact osteotomized fracture lines, the exact composition of the osteosynthesis constructs, the fixation mode of the pelvis models in the testing machine and others, were not included in the analysis of this review. However, these details can have an influence on the biomechanical test properties and must also be considered when interpreting or designing a biomechanical test setup.

Fourthly, this review did not include the results of the biomechanical tests and their interpretation as well as their clinical implications. As described above, it must be kept in mind, that the comparability and the clinical implications of the biomechanical studies reviewed are limited and should be critically evaluated.

## Conclusion

Biomechanical studies on fixation methods for acetabular fractures reported heterogeneous and partially inconsistent experimental setups, which were to some extent inadequately described or specified. While there were some commonly used biomechanical test setups, which were frequently referred to in the literature, there was no standardized approach to biomechanically investigate fixation methods for acetabular fractures. Consequently, it is difficult to compare the studies and their results, and manifest differences between studies investigating similar questions and implants may simply be due to differences in the experimental setup.

Furthermore, the clinical relevance of some experiments may be questioned, since some loading protocols (including e.g. loading force, loading direction, number of cycles) were not based on physiological characteristics in humans. Nevertheless, biomechanical in vitro studies will remain an approximation of the actual characteristics in vivo, as inclusion of all muscle forces, all joint reaction forces and the influence of fracture callus is utopian.

Since it is very important to biomechanically investigate different fixation methods in the field of acetabular fractures, a standardized biomechanical test setup should be established. This would help to improve the comparability between biomechanical studies, which evaluate only a small subset of available osteosynthesis constructs in each case. Furthermore, a well-considered standardized biomechanical test approach would ensure that a clinically relevant and realistic test setup will be used in future biomechanical analyses. This could improve the choice of osteosynthesis constructs, in order to achieve a better clinical outcome after surgery of acetabular fractures.

## Supplementary Information

Below is the link to the electronic supplementary material.

**Supplementary Table 1** Detailed listing of studies using a biomechanical test setup to investigate fixation constructs for acetabular fractures. **Supplementary Table 2** Detailed listing of studies using a finite element analysis to investigate fixation constructs for acetabular fractures (PDF 158 KB)

**Supplementary Table 3** PRISMA 2020 Checklist (PDF 85 KB)

## Data Availability

All data used and analyzed in this review are available within the article, its supplementary materials and the original publications. This review was not registered in PROSPERO because literature reviews that use a systematic search are not accepted for registration in PROSPERO. The complete review protocol is presented in the Materials and Methods section.

## References

[1] Laird A, Keating JF (2005). Acetabular fractures: a 16-year prospective epidemiological study. J Bone Jt Surg Br.

[2] Hanschen M, Pesch S, Huber-Wagner S, Biberthaler P (2017). Management of acetabular fractures in the geriatric patient. SICOT J.

[3] Mears DC (1999). Surgical treatment of acetabular fractures in elderly patients with osteoporotic bone. J Am Acad Orthop Surg.

[4] Rinne PP, Laitinen MK, Huttunen T, Kannus P, Mattila VM (2017). The incidence and trauma mechanisms of acetabular fractures: a nationwide study in Finland between 1997 and 2014. Injury.

[5] Boudissa M, Francony F, Kerschbaumer G, Ruatti S, Milaire M, Merloz P (2017). Epidemiology and treatment of acetabular fractures in a level-1 trauma centre: Retrospective study of 414 patients over 10 years. Orthop Traumatol Surg Res.

[6] Giannoudis PV, Grotz MR, Papakostidis C, Dinopoulos H (2005). Operative treatment of displaced fractures of the acetabulum. A meta-analysis. J Bone Jt Surg Br..

[7] Guerado E, Cano JR, Cruz E (2012). Fractures of the acetabulum in elderly patients: an update. Injury.

[8] Antell NB, Switzer JA, Schmidt AH (2017). Management of acetabular fractures in the elderly. J Am Acad Orthop Surg.

[9] Vipulendran K, Kelly J, Rickman M, Chesser T (2021). Current concepts: managing acetabular fractures in the elderly population. Eur J Orthop Surg Traumatol.

[10] Kelly J, Ladurner A, Rickman M (2020). Surgical management of acetabular fractures—a contemporary literature review. Injury.

[11] Matta JM (1996). Fractures of the acetabulum: accuracy of reduction and clinical results in patients managed operatively within three weeks after the injury. J Bone Jt Surg Am.

[12] Tannast M, Najibi S, Matta JM (2012). Two to twenty-year survivorship of the hip in 810 patients with operatively treated acetabular fractures. J Bone Jt Surg Am.

[13] Ochs BG, Marintschev I, Hoyer H, Rolauffs B, Culemann U, Pohlemann T (2010). Changes in the treatment of acetabular fractures over 15 years: analysis of 1266 cases treated by the German Pelvic Multicentre Study Group (DAO/DGU). Injury.

[14] Daurka JS, Pastides PS, Lewis A, Rickman M, Bircher MD (2014). Acetabular fractures in patients aged > 55 years: a systematic review of the literature. Bone Jt J..

[15] Boelch SP, Jordan MC, Meffert RH, Jansen H (2017). Comparison of open reduction and internal fixation and primary total hip replacement for osteoporotic acetabular fractures: a retrospective clinical study. Int Orthop.

[16] Mears DC, Velyvis JH, Chang CP (2003). Displaced acetabular fractures managed operatively: indicators of outcome. Clin Orthop Relat Res.

[17] Zha GC, Sun JY, Dong SJ (2013). Predictors of clinical outcomes after surgical treatment of displaced acetabular fractures in the elderly. J Orthop Res.

[18] Moed BR, Carr SE, Watson JT (2000). Open reduction and internal fixation of posterior wall fractures of the acetabulum. Clin Orthop Relat Res.

[19] Laflamme GY, Hebert-Davies J, Rouleau D, Benoit B, Leduc S (2011). Internal fixation of osteopenic acetabular fractures involving the quadrilateral plate. Injury.

[20] Ziran N, Soles GLS, Matta JM (2019). Outcomes after surgical treatment of acetabular fractures: a review. Patient Saf Surg.

[21] ISO 14602 (2010). Non-active surgical implants—implants for osteosynthesis—particular requirements.

[22] Gardner MJ, Silva MJ, Krieg JC (2012). Biomechanical testing of fracture fixation constructs: variability, validity, and clinical applicability. J Am Acad Orthop Surg.

[23] Page MJ, McKenzie JE, Bossuyt PM, Boutron I, Hoffmann TC, Mulrow CD (2021). The PRISMA 2020 statement: an updated guideline for reporting systematic reviews. BMJ.

[24] Page MJ, Moher D, Bossuyt PM, Boutron I, Hoffmann TC, Mulrow CD (2021). PRISMA 2020 explanation and elaboration: updated guidance and exemplars for reporting systematic reviews. BMJ.

[25] Letournel E (1980). Acetabulum fractures: classification and management. Clin Orthop Relat Res.

[26] Judet R, Judet J, Letournel E (1964). Fractures of the acetabulum: classification and surgical approaches for open reduction. Preliminary report. J Bone Jt Surg Am..

[27] Schorler H, Capanni F, Gaashan M, Wendlandt R, Jurgens C, Schulz AP (2017). Bone plates for osteosynthesis—a systematic review of test methods and parameters for biomechanical testing. Biomed Tech (Berl).

[28] Le Quang H, Schmoelz W, Lindtner RA, Schwendinger P, Blauth M, Krappinger D (2020). Biomechanical comparison of fixation techniques for transverse acetabular fractures—Single-leg stance vs. sit-to-stand loading. Injury.

[29] Moktar J, Machin A, Bougherara H, Schemitsch EH, Zdero R (2020). Biomechanical analysis of transverse acetabular fracture fixation in the elderly via the posterior versus the anterior approach with and without a total hip arthroplasty. Proc Inst Mech Eng H.

[30] Ryan W, Alfonso NA, Baldini T, Kumparatana P, Reiter M, Joyce C (2019). Precontoured quadrilateral surface acetabular plate fixation demonstrates increased stability when compared with pelvic reconstruction plates: a biomechanical study. J Orthop Trauma.

[31] Kistler BJ, Smithson IR, Cooper SA, Cox JL, Nayak AN, Santoni BG (2014). Are quadrilateral surface buttress plates comparable to traditional forms of transverse acetabular fracture fixation?. Clin Orthop Relat Res.

[32] Khajavi K, Lee AT, Lindsey DP, Leucht P, Bellino MJ, Giori NJ (2010). Single column locking plate fixation is inadequate in two column acetabular fractures. A biomechanical analysis. J Orthop Surg Res.

[33] Mehin R, Jones B, Zhu Q, Broekhuyse H (2009). A biomechanical study of conventional acetabular internal fracture fixation versus locking plate fixation. Can J Surg.

[34] Chang JK, Gill SS, Zura RD, Krause WR, Wang GJ (2001). Comparative strength of three methods of fixation of transverse acetabular fractures. Clin Orthop Relat Res.

[35] Becker CA, Kammerlander C, Cavalcanti Kussmaul A, Dotzauer F, Woiczinski M, Rubenbauer B (2018). Minimally invasive screw fixation is as stable as anterior plating in acetabular T-Type fractures - a biomechanical study. Orthop Traumatol Surg Res.

[36] Le Quang H, Schmoelz W, Lindtner RA, Dammerer D, Schwendinger P, Krappinger D (2021). Single column plate plus other column lag screw fixation vs. both column plate fixation for anterior column with posterior hemitransverse acetabular fractures—a biomechanical analysis using different loading protocols. Injury.

[37] Chen K, Yang F, Yao S, Xiong Z, Sun T, Guo X (2020). Biomechanical comparison of different fixation techniques for typical acetabular fractures in the elderly: the role of special quadrilateral surface buttress plates. J Bone Jt Surg Am.

[38] Busuttil T, Teuben M, Pfeifer R, Cinelli P, Pape HC, Osterhoff G (2019). Screw fixation of ACPHT acetabular fractures offers sufficient biomechanical stability when compared to standard buttress plate fixation. BMC Musculoskelet Disord.

[39] Tanoglu O, Alemdaroglu KB, Iltar S, Ozmeric A, Demir T, Erbay FK (2018). Biomechanical comparison of three different fixation techniques for anterior column posterior hemitransverse acetabular fractures using anterior intrapelvic approach. Injury.

[40] May C, Egloff M, Butscher A, Keel MJB, Aebi T, Siebenrock KA (2018). Comparison of fixation techniques for acetabular fractures involving the anterior column with disruption of the quadrilateral plate: a biomechanical study. J Bone Jt Surg Am.

[41] Spitler CA, Kiner D, Swafford R, Doty D, Goulet R, Jones LC (2017). Generating stability in elderly acetabular fractures—a biomechanical assessment. Injury.

[42] Uvarovas V, Satkauskas I, Urbonavicius R, Bucinskas V, Griskevicius J, Vengrauskas V (2016). Different stabilization techniques for type 62B3 acetabular fractures in combination with primary total hip arthroplasty in elderly patients: a biomechanical comparison. Geriatr Orthop Surg Rehabil.

[43] Zha GC, Sun JY, Dong SJ, Zhang W, Luo ZP (2015). A novel fixation system for acetabular quadrilateral plate fracture: a comparative biomechanical study. Biomed Res Int.

[44] Culemann U, Holstein JH, Kohler D, Tzioupis CC, Pizanis A, Tosounidis G (2010). Different stabilisation techniques for typical acetabular fractures in the elderly—a biomechanical assessment. Injury.

[45] Wu H, Song C, Shang R, Shao Q, Liu X, Zhang H (2020). Double column acetabular fractures fixation using a novel dynamic anterior plate-screw system: a biomechanical analysis. Injury.

[46] Wen X, Huang H, Wang C, Dong J, Lin X, Huang F (2020). Comparative biomechanical testing of customized three-dimensional printing acetabular-wing plates for complex acetabular fractures. Adv Clin Exp Med..

[47] Gillispie GJ, Babcock SN, McNamara KP, Dimoff ME, Aneja A, Brown PJ (2017). Biomechanical comparison of intrapelvic and extrapelvic fixation for acetabular fractures involving the quadrilateral plate. J Orthop Trauma.

[48] Wu YD, Cai XH, Liu XM, Zhang HX (2013). Biomechanical analysis of the acetabular buttress-plate: are complex acetabular fractures in the quadrilateral area stable after treatment with anterior construct plate-1/3 tube buttress plate fixation?. Clinics (Sao Paulo).

[49] Osterhoff G, Wulsten D, Babu S, Heyland M, Pari C (2019). Antegrade versus retrograde screw fixation of anterior column acetabular fractures: a biomechanical in vitro study. Eur J Trauma Emerg Surg.

[50] Marintschev I, Gras F, Schwarz CE, Pohlemann T, Hofmann GO, Culemann U (2012). Biomechanical comparison of different acetabular plate systems and constructs–the role of an infra-acetabular screw placement and use of locking plates. Injury.

[51] Gras F, Marintschev I, Schwarz CE, Hofmann GO, Pohlemann T, Culemann U (2012). Screw- versus plate-fixation strength of acetabular anterior column fractures: a biomechanical study. J Trauma Acute Care Surg.

[52] Su K, Liu S, Wu T, Yin Y, Zhang R, Li S (2017). Posterior column acetabular fracture fixation using a W-shaped angular plate: a biomechanical analysis. PLoS ONE.

[53] Marmor M, Knox R, Huang A, Herfat S (2020). Acetabulum cup stability in an early weight-bearing cadaveric model of geriatric posterior wall fractures. J Orthop Trauma.

[54] Pease F, Ward AJ, Stevenson AJ, Cunningham JL, Sabri O, Acharya M (2019). Posterior wall acetabular fracture fixation: a mechanical analysis of fixation methods. J Orthop Surg (Hong Kong).

[55] Altun G, Saka G, Demir T, Elibol FKE, Polat MO (2019). Precontoured buttress plate vs reconstruction plate for acetabulum posterior wall fractures: a biomechanical study. World J Orthop.

[56] Wu X (2018). A biomechanical comparison of different fixation techniques for fractures of the acetabular posterior wall. Int Orthop.

[57] Zhang Y, Tang Y, Wang P, Zhao X, Xu S, Zhang C (2013). Biomechanical comparison of different stabilization constructs for unstable posterior wall fractures of acetabulum. A cadaveric study. PLoS ONE.

[58] Xin-Wei L, Shuo-Gui X, Chun-Cai Z, Qing-Ge F, Pan-Feng W (2010). Biomechanical study of posterior wall acetabular fracture fixation using acetabular tridimensional memory alloy-fixation system. Clin Biomech (Bristol, Avon).

[59] Olson SA, Kadrmas MW, Hernandez JD, Glisson RR, West JL (2007). Augmentation of posterior wall acetabular fracture fixation using calcium-phosphate cement: a biomechanical analysis. J Orthop Trauma.

[60] Becker J, Winkler M, von Ruden C, Bliven E, Augat P, Resch H (2020). Comparison of two reinforcement rings for primary total hip arthroplasty addressing displaced acetabular fractures: a biomechanical analysis. Arch Orthop Traum Su.

[61] Kacira BK, Ozkaya M, Kiran U, Turkmen F, Arazi M, Demir T (2016). Biomechanical fixation strength comparison of ilioingunial and medial stoppa approaches on anterior column fractures. J Mech Med Biol..

[62] Shim VB, Boshme J, Vaitl P, Josten C, Anderson IA (2011). An efficient and accurate prediction of the stability of percutaneous fixation of acetabular fractures with finite element simulation. J Biomech Eng.

[63] Aziz MSR, Dessouki O, Samiezadeh S, Bougherara H, Schemitsch EH, Zdero R (2017). Biomechanical analysis using FEA and experiments of a standard plate method versus three cable methods for fixing acetabular fractures with simultaneous THA. Med Eng Phys.

[64] Yildirim AO, Alemdaroglu KB, Yuksel HY, Oken OF, Ucaner A (2015). Finite element analysis of the stability of transverse acetabular fractures in standing and sitting positions by different fixation options. Injury.

[65] Fan Y, Lei J, Zhu F, Li Z, Chen W, Liu X (2015). Biomechanical analysis of the fixation system for T-shaped acetabular fracture. Comput Math Methods Med.

[66] Lei J, Dong P, Li Z, Zhu F, Wang Z, Cai X (2017). Biomechanical analysis of the fixation systems for anterior column and posterior hemi-transverse acetabular fractures. Acta Orthop Traumatol Turc.

[67] Lei J, Liu H, Li Z, Wang Z, Liu X, Zhao L (2016). Biomechanical comparison of fixation systems in posterior wall fracture of acetabular by finite element analysis. Comput Assist Surg (Abingdon).

[68] Kocsis A, Varadi K, Szalai G, Kovacs T, Bodzay T (2019). Hybrid solution combining osteosynthesis and endoprosthesis for double column acetabular fractures in the elderly provide more stability with finite element model. Eklem Hastalik Cerrahisi.

[69] Yucens M, Alemdaroglu KB, Ozmeric A, Iltar S, Yildirim AO, Aydogan NH (2019). A comparative biomechanical analysis of suprapectineal and infrapectineal fixation on acetabular anterior column fracture by finite element modeling. Turk J Med Sci.

[70] Terzini M, Di Pietro A, Aprato A, Artiaco S, Masse A, Bignardi C (2021). Are suprapectineal quadrilateral surface buttressing plates performances superior to traditional fixation? A finite element analysis. Appl Sci-Basel..

[71] Liu XM, Huang JC, Wang GD, Lan SH, Wang HS, Pan CW (2016). Anterior titanium plate plus screw of square area combined with posterior column screw for the treatment of fracture of acetabulum involving square area. Int J Clin Exp Med.

[72] Bergmann G, Deuretzbacher G, Heller M, Graichen F, Rohlmann A, Strauss J (2001). Hip contact forces and gait patterns from routine activities. J Biomech.

[73] Bergmann G, Graichen F, Rohlmann A (1993). Hip joint loading during walking and running, measured in two patients. J Biomech.

[74] Witte H, Eckstein F, Recknagel S (1997). A calculation of the forces acting on the human acetabulum during walking. Based on in vivo force measurements, kinematic analysis and morphometry. Acta Anat (Basel).

[75] Gao YC, Guo Z, Fu J, Tian WJ (2011). Three-dimensional finite element analysis of the pelvis in sitting stance. J Clin Rehabil Tissue Eng Res.

[76] Bergmann G, Bender A, Dymke J, Duda G, Damm P (2016). Standardized loads acting in hip implants. PLoS ONE.

[77] Ferguson TA, Patel R, Bhandari M, Matta JM (2010). Fractures of the acetabulum in patients aged 60 years and older: an epidemiological and radiological study. J Bone Jt Surg Br.

[78] Mauffrey C, Hao J, Cuellar DO, Herbert B, Chen X, Liu B (2014). The epidemiology and injury patterns of acetabular fractures: are the USA and China comparable?. Clin Orthop Relat Res.

[79] Hammad AS, El-Khadrawe TA, Waly AH, Abu-Sheasha GA (2017). The efficacy of posterior plating and anterior column screw fixation in the management of T-shaped acetabular fractures—CART analysis of prospective cohort study. Injury.

[80] Suzuki T, Smith WR, Mauffrey C, Morgan SJ (2013). Safe surgical technique for associated acetabular fractures. Patient Saf Surg.

[81] Krappinger D, Schwendinger P, Lindtner RA (2019). Fluoroscopically guided acetabular posterior column screw fixation via an anterior approach. Oper Orthop Traumatol.

[82] Kumar A, Shah NA, Kershaw SA, Clayson AD (2005). Operative management of acetabular fractures. A review of 73 fractures. Injury.

[83] Helfet DL, Borrelli J, DiPasquale T, Sanders R (1992). Stabilization of acetabular fractures in elderly patients. J Bone Jt Surg Am.

[84] Pagenkopf E, Grose A, Partal G, Helfet DL (2006). Acetabular fractures in the elderly: treatment recommendations. HSS J.

[85] Gänsslen A, Müller M, Nerlich M, Lindahl J (2018). Acetabular fractures—diagnosis, indications, treatment strategies.

[86] Capone A, Peri M, Mastio M (2017). Surgical treatment of acetabular fractures in the elderly: a systematic review of the results. EFORT Open Rev.

[87] Olson SA, Marsh JL, Anderson DD, Latta Pe LL (2012). Designing a biomechanics investigation: choosing the right model. J Orthop Trauma.

[88] Cartner JL, Hartsell ZM, Ricci WM, Tornetta P (2011). Can we trust ex vivo mechanical testing of fresh–frozen cadaveric specimens? The effect of postfreezing delays. J Orthop Trauma.

[89] Zaffagnini S, Signorelli C, Bonanzinga T, Lopomo N, Raggi F, Roberti Di Sarsina T (2016). Soft tissues contribution to hip joint kinematics and biomechanics. Hip Int.

[90] Bsat S, Frei H, Beaule PE (2016). The acetabular labrum: a review of its function. Bone Jt J..

[91] Duarte RJ, Ramos A, Completo A, Relvas C, Simoes JA (2015). The importance of femur/acetabulum cartilage in the biomechanics of the intact hip: experimental and numerical assessment. Comput Methods Biomech Biomed Eng.

[92] Smith MV, Costic RS, Allaire R, Schilling PL, Sekiya JK (2014). A biomechanical analysis of the soft tissue and osseous constraints of the hip joint. Knee Surg Sports Traumatol Arthrosc.

[93] Fensky F, Weiser L, Sellenschloh K, Vollmer M, Hartel MJ, Morlock MM (2021). Biomechanical analysis of anterior pelvic ring fractures with intact peripelvic soft tissues: a cadaveric study. Eur J Trauma Emerg Surg.

[94] Cristofolini L, Viceconti M, Cappello A, Toni A (1996). Mechanical validation of whole bone composite femur models. J Biomech.

[95] Gardner MP, Chong AC, Pollock AG, Wooley PH (2010). Mechanical evaluation of large-size fourth-generation composite femur and tibia models. Ann Biomed Eng.

[96] Grover P, Albert C, Wang M, Harris GF (2011). Mechanical characterization of fourth generation composite humerus. Proc Inst Mech Eng H.

[97] F1839-08 (2021). Standard specification for rigid polyurethane foam for use as a standard material for testing orthopedic devices and instruments.

[98] Girardi BL, Attia T, Backstein D, Safir O, Willett TL, Kuzyk PR (2016). Biomechanical comparison of the human cadaveric pelvis with a fourth generation composite model. J Biomech.

[99] Ziran BH, Sharkey NA, Smith TS, Wang G, Chapman MW (1997). Modified transverse locking nail fixation of proximal femoral fractures. Clin Orthop Relat Res.

[100] Anderson AE, Peters CL, Tuttle BD, Weiss JA (2005). Subject-specific finite element model of the pelvis: development, validation and sensitivity studies. J Biomech Eng.

[101] Fetto JF (2019). A dynamic model of hip joint biomechanics: the contribution of soft tissues. Adv Orthop.

[102] Warrener AG (2017). Hominin hip biomechanics: changing perspectives. Anat Rec (Hoboken).

[103] Olson SA, Bay BK, Hamel A (1997). Biomechanics of the hip joint and the effects of fracture of the acetabulum. Clin Orthop Relat Res.

[104] Hind K, Hayes L, Basterfield L, Pearce MS, Birrell F (2020). Objectively-measured sedentary time, habitual physical activity and bone strength in adults aged 62 years: the Newcastle Thousand Families Study. J Public Health (Oxf).

[105] Schlaff RA, Baruth M, Boggs A, Hutto B (2017). Patterns of sedentary behavior in older adults. Am J Health Behav.

[106] Gine-Garriga M, Sansano-Nadal O, Tully MA, Caserotti P, Coll-Planas L, Rothenbacher D (2020). Accelerometer-measured sedentary and physical activity time and their correlates in European older adults: the SITLESS study. J Gerontol A Biol Sci Med Sci.

[107] Inai T, Takabayashi T, Edama M, Kubo M (2018). Effect of hip joint angle at seat-off on hip joint contact force during sit-to-stand movement: a computer simulation study. Biomed Eng Online.

[108] Millington PJ, Myklebust BM, Shambes GM (1992). Biomechanical analysis of the sit-to-stand motion in elderly persons. Arch Phys Med Rehabil.

[109] Pedersen DR, Brand RA, Davy DT (1997). Pelvic muscle and acetabular contact forces during gait. J Biomech.

[110] Greenwald AS, O'Connor JJ (1971). The transmission of load through the human hip joint. J Biomech.

[111] Taylor SJ, Walker PS (2001). Forces and moments telemetered from two distal femoral replacements during various activities. J Biomech.

[112] Brand RA, Pedersen DR, Davy DT, Kotzar GM, Heiple KG, Goldberg VM (1994). Comparison of hip force calculations and measurements in the same patient. J Arthroplasty.

[113] Bergmann G, Graichen F, Rohlmann A, Bender A, Heinlein B, Duda GN (2010). Realistic loads for testing hip implants. Biomed Mater Eng.

[114] Crowninshield RD, Johnston RC, Andrews JG, Brand RA (1978). A biomechanical investigation of the human hip. J Biomech.

[115] Polkowski GG, Clohisy JC (2010). Hip biomechanics. Sports Med Arthrosc Rev.

[116] Akyuz E, Braun JT, Brown NA, Bachus KN (2006). Static versus dynamic loading in the mechanical modulation of vertebral growth. Spine (Phila Pa 1976).

[117] Masuoka K, Michalek AJ, MacLean JJ, Stokes IA, Iatridis JC (2007). Different effects of static versus cyclic compressive loading on rat intervertebral disc height and water loss in vitro. Spine (Phila Pa 1976).

[118] Thornton GM, Schwab TD, Oxland TR (2007). Cyclic loading causes faster rupture and strain rate than static loading in medial collateral ligament at high stress. Clin Biomech (Bristol, Avon).

[119] F384-17 (2017). Standard specifications and test methods for metallic angled orthopedic fracture fixation devices.

[120] Bottlang M, Doornink J, Lujan TJ, Fitzpatrick DC, Marsh JL, Augat P (2010). Effects of construct stiffness on healing of fractures stabilized with locking plates. J Bone Jt Surg Am.

[121] Doebele S, Siebenlist S, Vester H, Wolf P, Hagn U, Schreiber U (2012). New method for detection of complex 3D fracture motion–verification of an optical motion analysis system for biomechanical studies. BMC Musculoskelet Disord.

[122] Motel C, Wichmann M, Matta RE (2019). 3D-optical measurement of implant biomechanics. Clin Oral Implants Res.

[123] Herzog W, Binder MD, Hirokawa N, Windhorst U (2009). Measurement techniques (biomechanics). Encyclopedia of neuroscience.

[124] Moazen M, Jones AC, Jin Z, Wilcox RK, Tsiridis E (2011). Periprosthetic fracture fixation of the femur following total hip arthroplasty: a review of biomechanical testing. Clin Biomech (Bristol, Avon).

