# Supplementary material for: Biomechanical analysis of fixation methods in acetabular fractures: a systematic review of test setups
Source: Eur J Trauma Emerg Surg. 2022 Mar 19;48(5):3541–60. doi: 10.1007/s00068-022-01936-9 (PMC9532317; doi:10.1007/s00068-022-01936-9)
Supplement: Supplementary file 1 — Supplementary Table 1 Detailed listing of studies using a biomechanical test setup to investigate fixation constructs for acetabular fractures. Supplementary Table 2 Detailed listing of studies using a finite element analysis to investigate fixation constructs for acetabular fractures (PDF 158 KB) [file 68_2022_1936_MOESM1_ESM.pdf]

**Supplementary Table 1** Detailed listing of studies using a biomechanical test setup to investigate fixation constructs for acetabular fractures. Reported characteristics for each category are described in the table cells. The studies are primarily sorted for tested fracture type and secondly sorted for year of publication.

| Paper | Fracture type | Material type | Fixation method investigated | Loading direction | Loading protocol and loading force | Measure-ment method | Outcome parameters |
|---|---|---|---|---|---|---|---|
| Su et al. 2017 [1] | Posterior column fracture | Cadaveric | Plate<br>Screw | Double limb stance | Cyclic loading up to 1400 N | Integrated displacement sensor<br>Strain gauge | Displacement (femoral head subluxation [mm])<br>Stiffness [N/mm]<br>Strain distribution/ Deformation [με] |
| Marmor et al. 2020 [2] | Posterior wall fracture | Cadaveric | Plate<br>THA | Direction of 12° in coronal plain and 36° in axial plane (comparable with mediosuperior direction described by Bergmann et al.) | Cyclic loading with up to 1.8 x BW | Optical measurement system<br>Integrated displacement sensor | Displacement (femoral head subluxation [mm]) |
| Pease et al. 2019 [3] | Posterior wall fracture | Synthetic | Plate<br>Screw | Plane of encompassing anterior superior iliac spine and symphysis pubis is vertical; joint surface of symphysis is vertical at 90° to this plane; 30° flexion is generated during loading by an angle plate | Cyclic loading with 1000 - 1500 N | Optical measurement system | Displacement (interfragmentary motion [mm])<br>Stiffness [N/mm] |
| Altun et al. 2019 [4] | Posterior wall fracture | Synthetic | Plate | Single-leg stance | Quasi-static loading with up to 2300 N<br>Cyclic loading with up to 2300 N | Optical measurement system | Displacement (gap motion [mm])<br>Stiffness [N/mm] |
| Wu et al. 2018 [5] | Posterior wall fracture | Synthetic | Plate<br>Screw | Femur prosthesis loaded with a flexion 75° in hip joint | Cyclic loading with 350 N<br>Loading to failure | Optical measurement system | Displacement (gap motion [mm])<br>Stiffness [N/mm]<br>Force at construct failure [N] |

| | | | | | | | |
|---|---|---|---|---|---|---|---|
| Zhang et al. 2013 [6] | Posterior wall fracture | Cadaveric | Plate<br>Screw | Double-limb stance | Cyclic loading up to 1500 N | Ultrasound-based system | Displacement (interfragmentary motion, vector dislocation [mm])<br>Stiffness [not specified] |
| Shim et al. 2011 [7] | Posterior wall fracture | Synthetic | Plate<br>Screw | Single-leg stance | Cyclic loading up to 900 N | Optical measurement system | Displacement (gap motion, interfragmentary motion [mm]) |
| Xin-wei et al. 2010 [8] | Posterior wall fracture | Cadaveric | Plate<br>Acetabular tridimensional memory alloy-fixation system (ATMFS) | Double-limb stance | Quasi-static loading up to 4000 N | Pressure-sensitive film | Contact area [$cm^2$]<br>Load and mean/peak pressure distribution within acetabulum [N, MPa] |
| Olson et al. 2007 [9] | Posterior wall fracture | Cadaveric | Plate<br>Calcium phosphate cement | Single leg stance via a simulated abductor load | Quasi-static loading up to 1700 - 2200 N | Pressure-sensitive film<br>Mechanical distance indicator | Fracture displacement (interfragmentary motion [μm])<br>Contact area [$cm^2$]<br>Load and mean/peak pressure distribution within acetabulum [N, MPa] |
| Osterhoff et al. 2019 [10] | Anterior column fracture | Synthetic | Screw | Mediosuperior direction | Cyclic loading with 25 - 750 N<br>Load to failure | Optical measurement system | Displacement (interfragmentary motion [mm])<br>Stiffness [N/mm]<br>Force and cycles at construct failure [N, n]<br>Failure energy [N*cycles]<br>Mode of failure |
| Gillispie et al 2017 [11] | Anterior column fracture with quadrilateral plate involvement | Cadaveric | Plate<br>Screw | Perpendicular to acetabulum | Quasi-static loading up to 300 N<br>Load to failure | Integrated displacement sensor | Displacement (femoral head subluxation [mm])<br>Stiffness [N/mm]<br>Force and Stiffness at construct failure [N, N/mm]<br>Elastic/plastic energy [kJ]<br>Yield/maximum force [N] |
| Kacira et al. 2016 [12] | Anterior column fracture | Synthetic | Plate | Double-limb stance | Quasi-static loading up to 2300 N | Optical measurement system | Displacement (interfragmentary motion [mm], femoral head subluxation [mm and °]) |
| Marintschev et al. 2012 [13] | High anterior column fracture | Synthetic | Plate<br>Screw | Single-leg stance with mobile pelvis | Cyclic loading with up to 800 N | Ultrasound-based system | Displacement (interfragmentary motion, vector dislocation [mm]) |

| | | | | | | | |
|---|---|---|---|---|---|---|---|
| Gras et al. 2012 [14] | High anterior column fracture | Synthetic | Plate<br>Screw | Single leg stance with mobile pelvis | Cyclic loading with 800 N<br>Load to failure | Ultrasound-based system | Displacement (interfragmentary motion, vector dislocation [mm])<br>Force at construct failure [N] |
| Le Quang et al. 2020 [15] | Transverse fracture | Synthetic | Plate<br>Screw | Single-leg stance<br>Sit-to-stand | Quasi-static loading up to 750 N | Optical measurement system | Displacement (gap motion [mm], interfragmentary rotation [°]) |
| Moktar et al. 2020 [16] | Transverse fracture | Synthetic | Plate<br>Screw<br>THA | Mediosuperior direction (45° superomedial and 15° posterior direction) | Quasi-static loading up to 750 N<br>Load to failure | Optical measurement system | Displacement (gap motion and sliding [mm])<br>Stiffness [N/mm]<br>Force at construct failure [N] |
| Ryan et al. 2019 [17] | Transverse fracture | Synthetic | Plate<br>Screw | Mediosuperior direction (40° superomedial direction) | Cyclic loading with 1700 N<br>Load to failure with 4800 N cyclically | Optical measurement system | Displacement (gap motion [mm])<br>Stiffness [N/mm]<br>Cycles at construct failure [n] |
| Kistler et al. 2014 [18] | Transverse fracture | Synthetic | Plate<br>Screw | Mediosuperior direction | Cyclic loading with 17.5 - 1750 N<br>Load to failure | Optical measurement system | Displacement (gap motion [mm])<br>Stiffness [N/mm]<br>Force at construct failure [N] |
| Khajavi et al. 2010 [19] | Transverse fracture | Synthetic | Plate<br>Screw | Mediosuperior direction (45° superomedial and 25° posterior direction) | Quasi-static loading with up to 2000 N | Optical measurement system | Displacement (gap motion [mm])<br>Stiffness [N/mm] |
| Mehin et al. 2009 [20] | Transverse fracture | Cadaveric | Plate<br>Screw | Perpendicular to acetabulum | Cyclic loading with 50 - 250 N<br>Load to failure | Optical measurement system | Displacement (gap motion [mm])<br>Stiffness [N/mm]<br>Force at construct failure [N] |
| Chang et al. 2001 [21] | Transverse fracture | Cadaveric | Plate<br>Screw<br>Wire | Mediosuperior direction (45° superomedial and 15° posterior direction) | Load to failure | Integrated displacement sensor<br>Strain gauge | Stiffness [MPa]<br>Stress distribution [MPa]<br>Yield/maximum strength [MPa] |
| Becker et al. 2018 [22] | T-shaped fracture | Synthetic | Plate<br>Screw<br>INFIX (subcutaneous internal anterior fixation) | Single-leg stance | Cyclic loading with 150 - 250 N<br>Quasi-static loading up to 600 N | Ultrasound-based system | Displacement (interfragmentary motion [mm])<br>Stiffness [N/mm] |
| Le Quang et al. 2021 [23] | Anterior column posterior hemitransverse fracture | Synthetic | Plate<br>Screw | Single-leg stance<br>Sit-to-stand | Quasi-static loading with 50 - 750 N | Optical measurement system | Displacement (gap motion [mm], interfragmentary rotation [°]) |

| | | | | | | | |
|---|---|---|---|---|---|---|---|
| Becker et al. 2020 [24] | Anterior column posterior hemitransverse fracture | Synthetic | THA | Single-leg stance with mobile pelvis and angles comparable to Bergmann et al. | Cyclic loading with 250 - 2400 N | Optical measurement system | Displacement (gap motion [mm], interfragmentary rotation [°]) |
| Chen et al. 2020 [25] | Anterior column posterior hemitransverse fracture | Synthetic | Plate<br>Screw | Mediosuperior direction | Cyclic loading with 35 - 750 N | Optical measurement system | Displacement (interfragmentary motion [mm])<br>Stiffness [N/mm] |
| Busuttil et al. 2019 [26] | Anterior column posterior hemitransverse fractures | Synthetic | Plate<br>Screw | Mediosuperior direction | Cyclic loading with 17.5 - 2450 N<br>Load to failure | Optical measurement system | Displacement (gap motion [mm], interfragmentary rotation [°])<br>femoral head subluxation [mm]<br>Cycles and force at construct failure [N, n]<br>Failure energy [N*cycles] |
| Tanoglu et al. 2018 [27] | Anterior column posterior hemitransverse fractures | Synthetic | Plate<br>Screw | Single-leg stance | Cyclic loading with 50 - 500 N<br>Quasi-static loading with up to 1200 N | Optical measurement system | Displacement (gap motion [mm])<br>Stiffness [N/mm] |
| May et al. 2018 [28] | Anterior column posterior hemitransverse fracture | Synthetic | Plate<br>Screw | Point of maximum vertical hip contact force during walking as defined by Bergmann et al.<br>Not further specified | Cyclic loading with 35 - 350 N<br>Load to failure | Optical measurement system | Displacement (gap motion [mm])<br>Force at construct failure [N] |
| Aziz et al. 2017 [29] | Anterior column posterior hemitransverse fracture | Synthetic | Plate<br>Cable fixation<br>THA | Mediosuperior direction (45° superomedial and 20° posterior direction) | Quasi-static loading up to 2207 N<br>Load to failure | Optical measurement system | Displacement (gap motion and sliding [mm])<br>Stiffness [N/mm]<br>Force at construct failure [N]<br>Failure energy [J] |
| Spitler et al. 2017 [30] | Anterior column posterior hemitransverse fracture | Synthetic | Plate<br>Screw | Mediosuperior direction (45° superomedial and 15° posterior direction) | Quasi-static loading with 50 - 1050 N | Optical measurement system | Displacement (interfragmentary motion [mm])<br>Stiffness [N/mm] |
| Uvarovas et al. 2016 [31] | Anterior column posterior hemitransverse fracture | Synthetic | Plate<br>THA | Single-leg stance | Quasi-static loading with up to 1187 N | Mechanical distance indicators | Displacement (gap motion [mm]) |
| Zha et al. 2015 [32] | Anterior column posterior hemitransverse fracture | Synthetic | Plate<br>Screw | Perpendicular | Quasi-static loading up to 300 N<br>Load to failure | Integrated displacement sensor | Displacement (not specified [mm])<br>Stiffness [N/mm] |

| | | | | | | | |
|---|---|---|---|---|---|---|---|
| | | | AFRIF (acetabular fracture reduction internal fixator) | | | | Force at construct failure [N] |
| Culemann et al. 2010 [33] | Anterior column posterior hemitransverse fracture | Synthetic<br>Cadaveric | Plate<br>Screw | Single-leg stance with mobile pelvis | Quasi-static loading with 750 N (synthetic) or 375 N (cadaveric) | Ultrasound-based system | Displacement (interfragmentary motion [mm]) |
| Wu et al. 2020 [34] | High associated both column fracture | Cadaveric | Plate<br>Screw | Double-limb stance | Quasi-static loading up to 800N | Mechanical (digital) distance indicator<br>Beam sensor | Displacement (interfragmentary motion, longitudinal motion of pelvis [mm])<br>Stiffness [N/mm] |
| Wen et al. 2020 [35] | Associated both column fracture | Cadaveric | Plate | Perpendicular to acetabulum | Quasi-static loading up to 700 N<br>Load to failure | Optical measurement system | Displacement (gap motion [mm])<br>Stiffness [N/mm]<br>Force at construct failure [N] |
| Wu et al. 2013 [36] | High associated both column fracture | Cadaveric | Plate | Double-limb stance<br>Sitting position | Cyclic loading with 400 - 700 N | Mechanical (digital) distance indicator<br>Beam sensor | Displacement (interfragmentary motion, longitudinal motion of pelvis [mm])<br>Stiffness [N/mm] |

**Supplementary table 2** Detailed listing of studies using a finite element analysis to investigate fixation constructs for acetabular fractures. Reported characteristics for each category are described in the table cells. The studies are primarily sorted for tested fracture type and secondly sorted for year of publication.

| **Paper** | **Fracture type** | **Fixation method investigated** | **Loading direction** | **Loading protocol and loading force** | **Outcome parameters** |
|---|---|---|---|---|---|
| Lei et al. 2016 [37] | Posterior wall fracture | Plate<br>Screw | Double-limb stance loading<br>Sit-to-stand with various angles | Static loading with 600 - 1200 N | Displacement (along fracture lines [mm])<br>(Von Mises) Stress distribution [MPa] |
| Shim et al. 2011 [7] | Posterior wall fracture | Plate<br>Screw | Single-leg stance | Cyclic loading up to 900 N | Displacement (interfragmentary motion [mm]) |

| | | | | | |
|---|---|---|---|---|---|
| Yücens et al. 2019 [38] | Anterior column fracture | Plate<br>Screw | Double-limb stance | Static loading with 2300 N | Displacement (interfragmentary motion [mm])<br>(Von Mises) Stress distribution [MPa] |
| Terzini et al. 2021 [39] | Transverse fracture<br>T-shaped fracture | Plate<br>Screw | Single-leg stance with resultant forces according to Bergmann et al. | Static loading with 2032 N | (Von Mises) Stress distribution [MPa]<br>Displacement (interfragmentary motion [mm])<br>Axial and shear strain calculated from displacement [%] |
| Yildirim et al. 2015 [40] | Transverse fracture | Plate<br>Screw | Double-limb stance<br>Sitting position | Static loading with 400 N | Displacement (interfragmentary motion [mm])<br>(Von Mises) Stress distribution [not specified] |
| Fan et al. 2015 [41] | T-shaped fracture | Plate<br>Screw | Double-limb stance loading | Static loading with 600 N | Displacement (along fracture lines [mm])<br>Stiffness [N/mm]<br>(Von Mises) Stress distribution [MPa] |
| Lei et al. 2017 [42] | Anterior column posterior hemitransverse fracture | Plate<br>Screw | Double-limb stance loading | Static loading with 600 N | Displacement (along fracture lines [mm])<br>Stiffness [N/mm]<br>(Von Mises) Stress distribution [MPa] |
| Aziz et al. 2017 [29] | Anterior column posterior hemitransverse fracture | Plate<br>Cable fixation<br>THA | Mediosuperior direction (45° superomedial and 20° posterior direction) | Static loading with 2207 N | (Von Mises) Stress distribution [MPa] |
| Liu et al. 2016 [43] | Anterior column posterior hemitransverse fracture | Plate<br>Screw | Multi-part model of 5 different gait phases with differing loading directions | Static loading with a multi-part model of 5 different gait phases with differing resultant forces | Displacement (gap motion [mm]) |
| Kocsis et al. 2019 [44] | Associated both column fracture combined with a transversal component | Plate<br>THA | Double-limb stance<br>Sit-to-stand<br>Climbing stairs | Static loading (force not specified) | Displacement (shift [not specified])<br>(Von Mises) Stress distribution [MPa] |

## References:


1. Su K, Liu S, Wu T, Yin Y, Zhang R, Li S, et al. Posterior column acetabular fracture fixation using a W-shaped angular plate: A biomechanical analysis. PLoS One. 2017;12(11):e0187886. https://doi.org/10.1371/journal.pone.0187886
2. Marmor M, Knox R, Huang A, Herfat S. Acetabulum Cup Stability in an Early Weight-Bearing Cadaveric Model of Geriatric Posterior Wall Fractures. J Orthop Trauma. 2020;34(1):55-61. https://doi.org/10.1097/BOT.0000000000001627
3. Pease F, Ward AJ, Stevenson AJ, Cunningham JL, Sabri O, Acharya M, et al. Posterior wall acetabular fracture fixation: A mechanical analysis of fixation methods. J Orthop Surg (Hong Kong). 2019;27(3):2309499019859838. https://doi.org/10.1177/2309499019859838

4. Altun G, Saka G, Demir T, Elibol FKE, Polat MO. Precontoured buttress plate vs reconstruction plate for acetabulum posterior wall fractures: A biomechanical study. World J Orthop. 2019;10(5):219-27. https://doi.org/10.5312/wjo.v10.i5.219
5. Wu X. A biomechanical comparison of different fixation techniques for fractures of the acetabular posterior wall. Int Orthop. 2018;42(3):673-9. https://doi.org/10.1007/s00264-017-3728-3
6. Zhang Y, Tang Y, Wang P, Zhao X, Xu S, Zhang C. Biomechanical comparison of different stabilization constructs for unstable posterior wall fractures of acetabulum. A cadaveric study. PLoS One. 2013;8(12):e82993. https://doi.org/10.1371/journal.pone.0082993
7. Shim VB, Boshme J, Vaitl P, Josten C, Anderson IA. An efficient and accurate prediction of the stability of percutaneous fixation of acetabular fractures with finite element simulation. J Biomech Eng. 2011;133(9):094501. https://doi.org/10.1115/1.4004821
8. Xin-Wei L, Shuo-Gui X, Chun-Cai Z, Qing-Ge F, Pan-Feng W. Biomechanical study of posterior wall acetabular fracture fixation using acetabular tridimensional memory alloy-fixation system. Clin Biomech (Bristol, Avon). 2010;25(4):312-7. https://doi.org/10.1016/j.clinbiomech.2010.01.008
9. Olson SA, Kadrmas MW, Hernandez JD, Glisson RR, West JL. Augmentation of posterior wall acetabular fracture fixation using calcium-phosphate cement: a biomechanical analysis. J Orthop Trauma. 2007;21(9):608-16. https://doi.org/10.1097/BOT.0b013e3181591397
10. Osterhoff G, Wulsten D, Babu S, Heyland M, Pari C. Antegrade versus retrograde screw fixation of anterior column acetabular fractures: a biomechanical in vitro study. Eur J Trauma Emerg Surg. 2019. 10.1007/s00068-019-01255-6. https://doi.org/10.1007/s00068-019-01255-6
11. Gillispie GJ, Babcock SN, McNamara KP, Dimoff ME, Aneja A, Brown PJ, et al. Biomechanical Comparison of Intrapelvic and Extrapelvic Fixation for Acetabular Fractures Involving the Quadrilateral Plate. J Orthop Trauma. 2017;31(11):570-6. https://doi.org/10.1097/BOT.0000000000000963
12. Kacira BK, Ozkaya M, Kiran U, Turkmen F, Arazi M, Demir T. Biomechanical Fixation Strength Comparison of Ilioingunial and Medial Stoppa Approaches on Anterior Column Fractures. J Mech Med Biol. 2016;16(6). https://doi.org/10.1142/S0219519416500810
13. Marintschev I, Gras F, Schwarz CE, Pohlemann T, Hofmann GO, Culemann U. Biomechanical comparison of different acetabular plate systems and constructs--the role of an infra-acetabular screw placement and use of locking plates. Injury. 2012;43(4):470-4. https://doi.org/10.1016/j.injury.2011.11.009
14. Gras F, Marintschev I, Schwarz CE, Hofmann GO, Pohlemann T, Culemann U. Screw- versus plate-fixation strength of acetabular anterior column fractures: a biomechanical study. J Trauma Acute Care Surg. 2012;72(6):1664-70. https://doi.org/10.1097/TA.0b013e3182463b45
15. Le Quang H, Schmoelz W, Lindtner RA, Schwendinger P, Blauth M, Krappinger D. Biomechanical comparison of fixation techniques for transverse acetabular fractures - Single-leg stance vs. sit-to-stand loading. Injury. 2020;51(10):2158-64. https://doi.org/10.1016/j.injury.2020.07.008
16. Moktar J, Machin A, Bougherara H, Schemitsch EH, Zdero R. Biomechanical analysis of transverse acetabular fracture fixation in the elderly via the posterior versus the anterior approach with and without a total hip arthroplasty. Proc Inst Mech Eng H. 2020;234(9):966-74. https://doi.org/10.1177/0954411920935759
17. Ryan W, Alfonso NA, Baldini T, Kumparatana P, Reiter M, Joyce C, et al. Precontoured Quadrilateral Surface Acetabular Plate Fixation Demonstrates Increased Stability When Compared With Pelvic Reconstruction Plates: A Biomechanical Study. J Orthop Trauma. 2019;33(9):e325-e30. https://doi.org/10.1097/BOT.0000000000001496
18. Kistler BJ, Smithson IR, Cooper SA, Cox JL, Nayak AN, Santoni BG, et al. Are quadrilateral surface buttress plates comparable to traditional forms of transverse acetabular fracture fixation? Clin Orthop Relat Res. 2014;472(11):3353-61. https://doi.org/10.1007/s11999-014-3800-x
19. Khajavi K, Lee AT, Lindsey DP, Leucht P, Bellino MJ, Giori NJ. Single column locking plate fixation is inadequate in two column acetabular fractures. A biomechanical analysis. J Orthop Surg Res. 2010;5:30. https://doi.org/10.1186/1749-799X-5-30
20. Mehin R, Jones B, Zhu Q, Broekhuyse H. A biomechanical study of conventional acetabular internal fracture fixation versus locking plate fixation. Can J Surg. 2009;52(3):221-8.
21. Chang JK, Gill SS, Zura RD, Krause WR, Wang GJ. Comparative strength of three methods of fixation of transverse acetabular fractures. Clin Orthop Relat Res. 2001. 10.1097/00003086-200111000-00057(392):433-41. https://doi.org/10.1097/00003086-200111000-00057
22. Becker CA, Kammerlander C, Cavalcanti Kussmaul A, Dotzauer F, Woiczinski M, Rubenbauer B, et al. Minimally invasive screw fixation is as stable as anterior plating in acetabular T-Type fractures - a biomechanical study. Orthop Traumatol Surg Res. 2018;104(7):1055-61. https://doi.org/10.1016/j.otsr.2018.06.013
23. Le Quang H, Schmoelz W, Lindtner RA, Dammerer D, Schwendinger P, Krappinger D. Single column plate plus other column lag screw fixation vs. both column plate fixation for anterior column with posterior hemitransverse acetabular fractures - a biomechanical analysis using different loading protocols. Injury. 2021. 10.1016/j.injury.2020.12.041. https://doi.org/10.1016/j.injury.2020.12.041
24. Becker J, Winkler M, von Ruden C, Bliven E, Augat P, Resch H. Comparison of two reinforcement rings for primary total hip arthroplasty addressing displaced acetabular fractures: a biomechanical analysis. Arch Orthop Traum Su. 2020;140(12):1947-54. https://doi.org/10.1007/s00402-020-03433-3

25. Chen K, Yang F, Yao S, Xiong Z, Sun T, Guo X. Biomechanical Comparison of Different Fixation Techniques for Typical Acetabular Fractures in the Elderly: The Role of Special Quadrilateral Surface Buttress Plates. J Bone Joint Surg Am. 2020;102(14):e81. https://doi.org/10.2106/JBJS.19.01027
26. Busuttil T, Teuben M, Pfeifer R, Cinelli P, Pape HC, Osterhoff G. Screw fixation of ACPHT acetabular fractures offers sufficient biomechanical stability when compared to standard buttress plate fixation. BMC Musculoskelet Disord. 2019;20(1):39. https://doi.org/10.1186/s12891-019-2422-6
27. Tanoglu O, Alemdaroglu KB, Iltar S, Ozmeric A, Demir T, Erbay FK. Biomechanical comparison of three different fixation techniques for anterior column posterior hemitransverse acetabular fractures using anterior intrapelvic approach. Injury. 2018;49(8):1513-9. https://doi.org/10.1016/j.injury.2018.06.020
28. May C, Egloff M, Butscher A, Keel MJB, Aebi T, Siebenrock KA, et al. Comparison of Fixation Techniques for Acetabular Fractures Involving the Anterior Column with Disruption of the Quadrilateral Plate: A Biomechanical Study. J Bone Joint Surg Am. 2018;100(12):1047-54. https://doi.org/10.2106/JBJS.17.00295
29. Aziz MSR, Dessouki O, Samiezadeh S, Bougherara H, Schemitsch EH, Zdero R. Biomechanical analysis using FEA and experiments of a standard plate method versus three cable methods for fixing acetabular fractures with simultaneous THA. Med Eng Phys. 2017;46:71-8. https://doi.org/10.1016/j.medengphy.2017.06.004
30. Spitler CA, Kiner D, Swafford R, Doty D, Goulet R, Jones LC, et al. Generating stability in elderly acetabular fractures-A biomechanical assessment. Injury. 2017;48(10):2054-9. https://doi.org/10.1016/j.injury.2017.07.020
31. Uvarovas V, Satkauskas I, Urbonavicius R, Bucinskas V, Griskevicius J, Vengrauskas V, et al. Different Stabilization Techniques for Type 62B3 Acetabular Fractures in Combination With Primary Total Hip Arthroplasty in Elderly Patients: A Biomechanical Comparison. Geriatr Orthop Surg Rehabil. 2016;7(3):153-7. https://doi.org/10.1177/2151458516658329
32. Zha GC, Sun JY, Dong SJ, Zhang W, Luo ZP. A novel fixation system for acetabular quadrilateral plate fracture: a comparative biomechanical study. Biomed Res Int. 2015;2015:391032. https://doi.org/10.1155/2015/391032
33. Culemann U, Holstein JH, Kohler D, Tzioupis CC, Pizanis A, Tosounidis G, et al. Different stabilisation techniques for typical acetabular fractures in the elderly--a biomechanical assessment. Injury. 2010;41(4):405-10. https://doi.org/10.1016/j.injury.2009.12.001
34. Wu H, Song C, Shang R, Shao Q, Liu X, Zhang H, et al. Double column acetabular fractures fixation using a novel dynamic anterior plate-screw system: A biomechanical analysis. Injury. 2020;52(3):407-13. https://doi.org/10.1016/j.injury.2020.10.066
35. Wen X, Huang H, Wang C, Dong J, Lin X, Huang F, et al. Comparative biomechanical testing of customized three-dimensional printing acetabular-wing plates for complex acetabular fractures. Adv Clin Exp Med. 2020;29(4):459-68. https://doi.org/10.17219/acem/116749
36. Wu YD, Cai XH, Liu XM, Zhang HX. Biomechanical analysis of the acetabular buttress-plate: are complex acetabular fractures in the quadrilateral area stable after treatment with anterior construct plate-1/3 tube buttress plate fixation? Clinics (Sao Paulo). 2013;68(7):1028-33. https://doi.org/10.6061/clinics/2013(07)22
37. Lei J, Liu H, Li Z, Wang Z, Liu X, Zhao L. Biomechanical comparison of fixation systems in posterior wall fracture of acetabular by finite element analysis. Comput Assist Surg (Abingdon). 2016;21(1):117-26. https://doi.org/10.1080/24699322.2016.1218052
38. Yucens M, Alemdaroglu KB, Ozmeric A, Iltar S, Yildirim AO, Aydogan NH. A comparative biomechanical analysis of suprapectineal and infrapectineal fixation on acetabular anterior column fracture by finite element modeling. Turk J Med Sci. 2019;49(1):442-8. https://doi.org/10.3906/sag-1806-72
39. Terzini M, Di Pietro A, Aprato A, Artiaco S, Masse A, Bignardi C. Are Suprapectineal Quadrilateral Surface Buttressing Plates Performances Superior to Traditional Fixation? A Finite Element Analysis. Appl Sci-Basel. 2021;11(2). https://doi.org/10.3390/app11020858
40. Yildirim AO, Alemdaroglu KB, Yuksel HY, Oken OF, Ucaner A. Finite element analysis of the stability of transverse acetabular fractures in standing and sitting positions by different fixation options. Injury. 2015;46 Suppl 2:S29-35. https://doi.org/10.1016/j.injury.2015.05.029
41. Fan Y, Lei J, Zhu F, Li Z, Chen W, Liu X. Biomechanical Analysis of the Fixation System for T-Shaped Acetabular Fracture. Comput Math Methods Med. 2015;2015:370631. https://doi.org/10.1155/2015/370631
42. Lei J, Dong P, Li Z, Zhu F, Wang Z, Cai X. Biomechanical analysis of the fixation systems for anterior column and posterior hemi-transverse acetabular fractures. Acta Orthop Traumatol Turc. 2017;51(3):248-53. https://doi.org/10.1016/j.aott.2017.02.003
43. Liu XM, Huang JC, Wang GD, Lan SH, Wang HS, Pan CW, et al. Anterior titanium plate plus screw of square area combined with posterior column screw for the treatment of fracture of acetabulum involving square area. Int J Clin Exp Med. 2016;9(1):108-19.
44. Kocsis A, Varadi K, Szalai G, Kovacs T, Bodzay T. Hybrid solution combining osteosynthesis and endoprosthesis for double column acetabular fractures in the elderly provide more stability with finite element model. Eklem Hastalik Cerrahisi. 2019;30(2):106-11. https://doi.org/10.5606/ehc.2019.66592

**Article title:** Biomechanical analysis of fixation methods in acetabular fractures – a systematic review of test setups

**Journal name:** European Journal of Trauma and Emergency Surgery

**Author names:** Nico Hinz [1], Julius Dehoust [1], Matthias Münch [2], Klaus Seidel [1,2], Tobias Barth [2], Arndt-Peter Schulz [1,3], Karl-Heinz Frosch [1,4] and Maximilian J. Hartel [1,4]*

**Affiliations:**
1 Department of Trauma, Orthopaedic Surgery and Sports Traumatology, BG Trauma Hospital Hamburg, Bergedorfer Strasse 10, 21033 Hamburg, Germany
2 Laboratory for Biomechanics, BG Trauma Hospital Hamburg, Bergedorfer Strasse 10, 21033 Hamburg, Germany
3 Fraunhofer Research Institution for Individualized and Cell-Based Medical Engineering, Mönkhofer Weg 239 a, 23562 Lübeck, Germany
4 Department of Trauma and Orthopaedic Surgery, University Medical Center Hamburg-Eppendorf, Martinistrasse 52, 20246 Hamburg, Germany

*** Corresponding author:** Maximilian J. Hartel; m.hartel@uke.de
